# Supplementary material for: Selective hydrogenolysis of catechyl lignin into propenylcatechol over an atomically dispersed ruthenium catalyst
Source: Nat Commun. 2021 Jan 18;12:416. doi: 10.1038/s41467-020-20684-1 (PMC7814062; doi:10.1038/s41467-020-20684-1)
Supplement: Supplementary file 1 — Supplementary Information [file 41467_2020_20684_MOESM1_ESM.pdf]

Supplementary Information for

# Selective Hydrogenolysis of Catechyl Lignin into Propenylcatechol over an Atomically Dispersed Ruthenium Catalyst

Shuizhong Wang[1], Kaili Zhang[1], Helong Li[1], Ling-Ping Xiao[2] & Guoyong Song[1*]

[1]Beijing Advanced Innovation Center for Tree Breeding by Molecular Design, Beijing Key Laboratory of Lignocellulosic Chemistry, Beijing Forestry University, Beijing, 100083 (P.R. China).

[*]email: songg@bjfu.edu.cn

[2]Center for Lignocellulosic Chemistry and Biomaterials, Dalian Polytechnic University, Dalian 116034 (P.R. China).

## Supplementary Methods

**XAFS measurements.** The Ru K-edge X-ray absorption data were collected at BL14W1 station in Shanghai Synchrotron Radiation Facility (SSRF, operated at 3.5 GeV with a maximum current of 250 mA). At BL14W1, the radiation was monochromatized by a Si (311) double-crystal monochromator. The intensity of the incident X-ray was monitored by an Ar-filled ion chamber (I0) in front of the sample. Solid samples were placed in an aluminum sample holder sealed with kapton tape. The data were collected as fluorescence excitation spectra with a Lytle detector. Energy was calibrated by the first peak maximum of the first derivative of a Ru foil (22117 eV), placed between two Ar-filled ionization chambers (I1 and I2) after the sample. The spectra of the references were recorded in transmission mode using an Ar-filled ionization chamber. All data were collected at room temperature.

**XAFS Data Analysis.** The acquired EXAFS data were processed according to the standard procedures using the Athena and Artemis implemented in the IFEFFIT software packages. The fitting detail is described below according to reported literature[1]:

The acquired EXAFS data were processed according to the standard procedures using the ATHENA module implemented in the IFEFFIT software packages. The EXAFS spectra were obtained by subtracting the post-edge background from the overall absorption and then normalizing with respect to the edge-jump step. Subsequently, the $\chi(k)$ data of were Fourier transformed to real (R) space using a hanning windows (dk=1.0 Å$^{-1}$) to separate the EXAFS contributions from different coordination shells. To obtain the quantitative structural parameters around central atoms, least-squares curve parameter fitting was performed using the ARTEMIS module of IFEFFIT software packages.[2]

$$\chi(k)=\sum_j \frac{N_j S_o^2 F_j(k)}{k R_j^2}\exp[-2k^2\sigma_j^2]\exp[\frac{-2R_j}{\lambda(k)}]\sin[2kR_j+\phi_j(k)]$$

**Supplementary Equation 1.** The equation used in EXAFS analysis.

$S_0^2$ is the amplitude reduction factor, $F_j(k)$ is the effective curved-wave backscattering amplitude, $N_j$ is the number of neighbors in the $j^{th}$ atomic shell, $R_j$ is the distance between the X-ray absorbing central atom and the atoms in the $j^{th}$ atomic shell (backscatterer), $\lambda$ is the mean free path in Å, $\phi_j(k)$ is the phase shift (including the phase shift for each shell and the total central

atom phase shift), $\sigma_j$ is the Debye-Waller parameter of the $j^{th}$ atomic shell (variation of distances around the average $R_j$). The functions $F_j(k)$, $\lambda$ and $\phi_j(k)$ were calculated with the ab initio code FEFF8.2. The coordination numbers of model samples were fixed as the nominal values. The obtained $S_0^2$ was fixed in the subsequent fitting. While the internal atomic distances $R$, Debye-Waller factor $\sigma^2$, and the edge-energy shift $\Delta E_0$ were allowed to run freely.

**Supplementary Table 1.** The results of ICP-AES and IC analysis.

| | ICP-AES analysis (wt%) | | |
|---|---|---|---|
| | Ru | Zn | Cl |
| Fresh Ru/ZnO/C | 0.2 | 51 | Not detected[b] |
| Spent Ru/ZnO/C[a] | 0.18 | 48 | |
| Liquid phase[a] | Not detected | Not detected | |

[a] Reaction conditions: C-lignin (50 mg), Ru/ZnO/C (15 mg, 30 wt%), MeOH (10 mL), 200 $^{o}$C, 3 MPa $H_2$, and 4 h. [b] based on XPS and ion chromatogram.

**Supplementary Note 1.** The spent catalyst (after 1$^{st}$ run) was collected by filtration, and was washed with DMSO and MeOH before ICP-AES analysis.

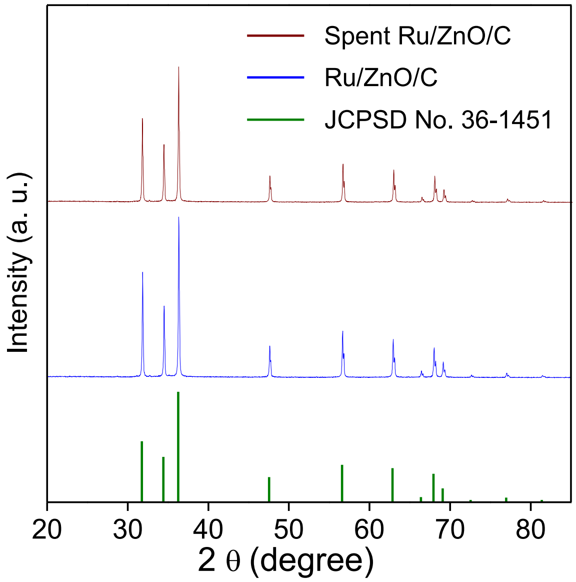


**Supplementary Figure 1.** XRD patterns of fresh and spent Ru/ZnO/C catalyst.

**Fresh catalyst**

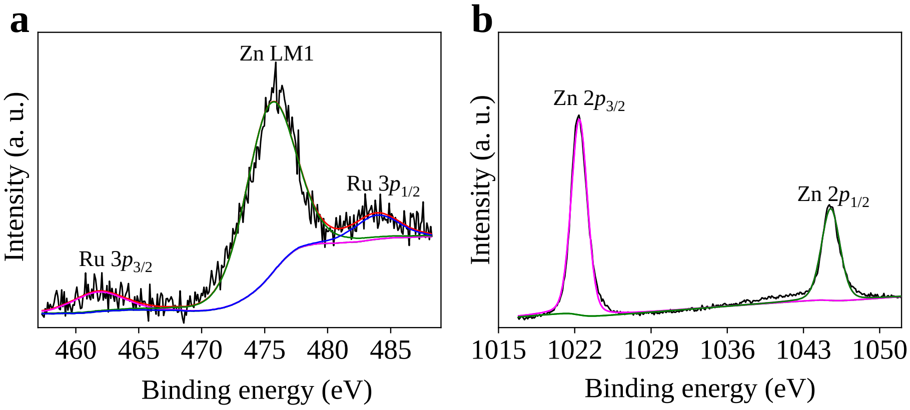


**Spent catalyst (after 1st run)**

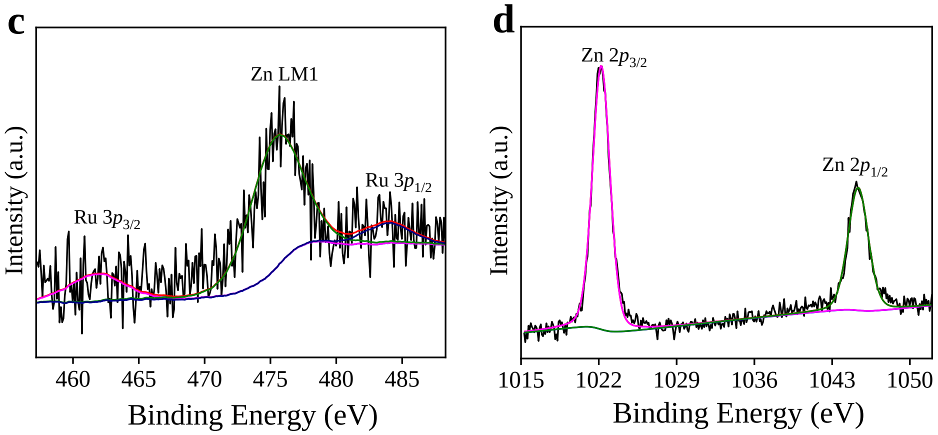


**Supplementary Figure 2.** XPS spectra of fresh (**a** & **b**) and spent (**c** & **d**) Ru/ZnO/C catalyst.

**Supplementary Note 2.** In Ru 3*p* spectra (**a** & **c**), fainter signals resonated at 462.1 and 484.2 eV were observed, which may be attributed to ruthenium oxide ($RuO_2$).

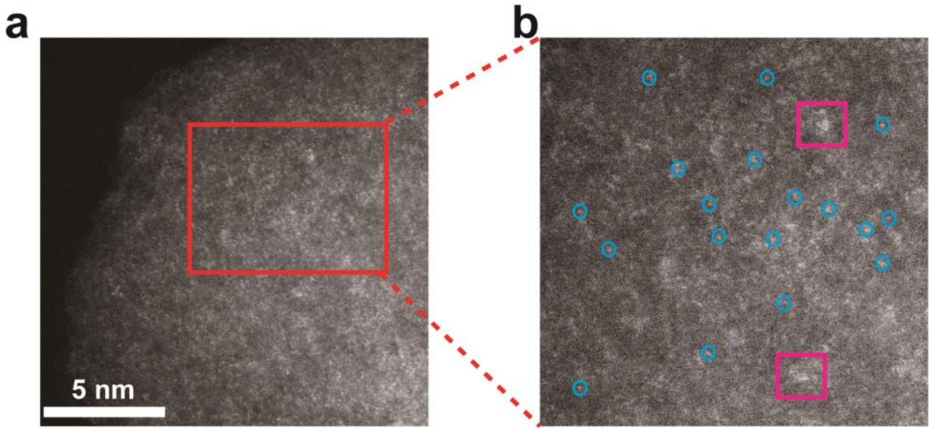


**Supplementary Figure 3. Characterizations of spent Ru/ZnO/C catalyst. a & b** HAADF-STEM images of spent Ru/ZnO/C catalyst after 1st use.

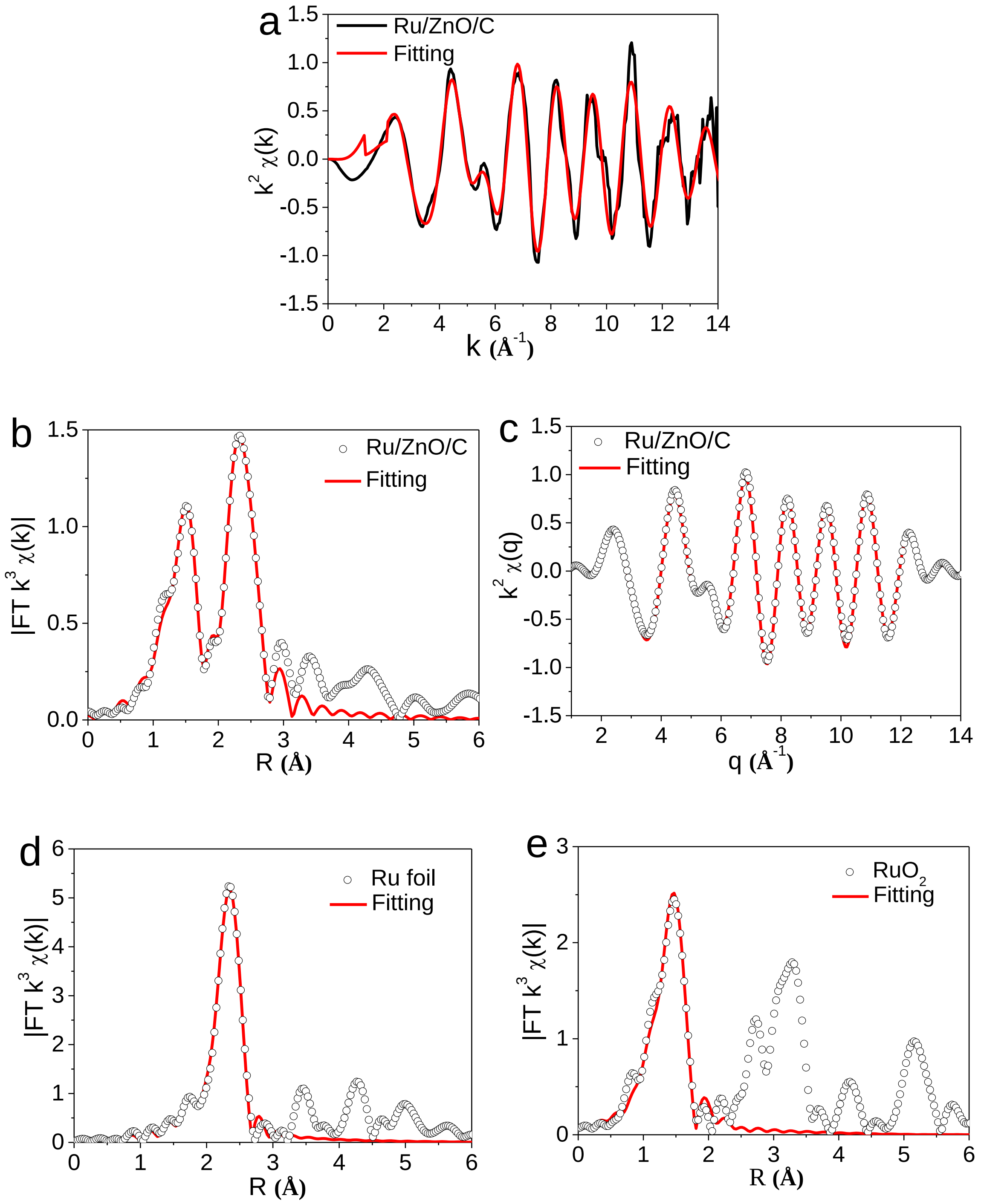


**Supplementary Figure 4.** EXAFS fitting curves. **a** k space EXAFS of Ru/ZnO/C. **b** FT-EXAFS of Ru/ZnO/C. **c** Inversed FT-EXAFS of Ru/ZnO/C catalyst. **d** FT-EXAFS of Ru foil. **e** FT-EXAFS of $RuO_2$.

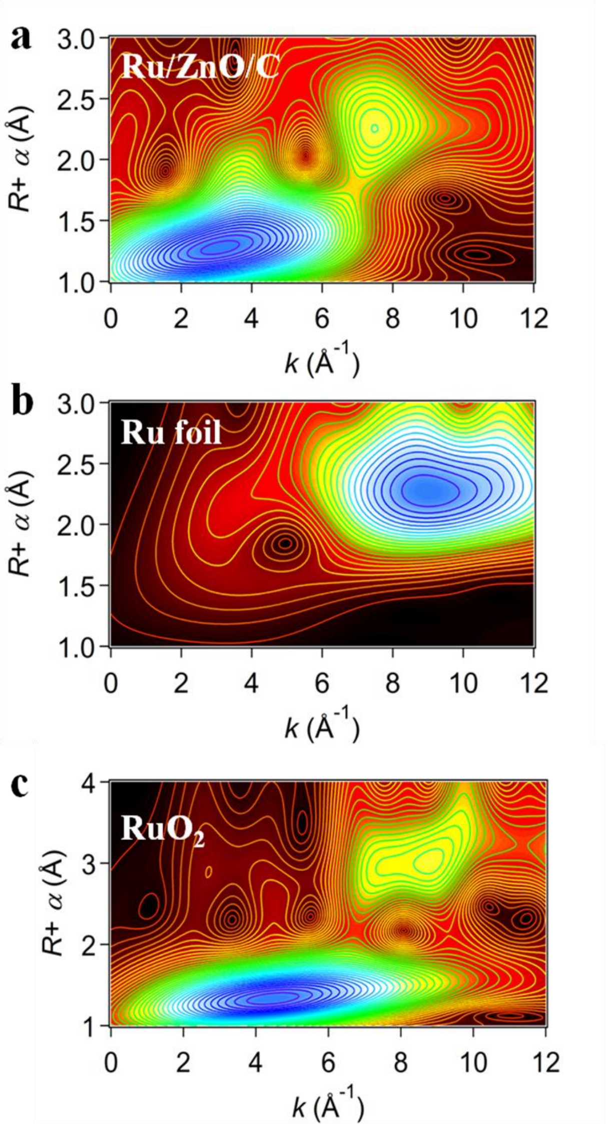


**Supplementary Figure 5.** Wavelet transformed (WT) $k^2$-weighted χ(k)-function of the Ru-K edge EXAFS spectra. **a** Ru/ZnO/C catalyst. **b** Ru foil. **c** $RuO_2$.

**Supplementary Table 2.** Structural parameters extracted from the Ru K-edge EXAFS fitting. ($S_0^2$=0.81)

| Sample | Scattering pair | CN | R(Å) | $\sigma^2$($10^{-3}$Å$^2$) | $\Delta E_0$(eV) | R factor |
|---|---|---|---|---|---|---|
| Ru/ZnO/C | Ru-O | 4.2 | 1.98 | 6.5 | 1.0 | 0.007 |
| | Ru-Ru | 6.7 | 2.66 | 6.8 | 2.0 | |
| Ru foil | Ru-Ru | 12* | 2.68 | 4.9 | 2.0 | 0.005 |
| $RuO_2$ | Ru-O | 6* | 1.98 | 4.6 | 1.0 | 0.006 |

**Supplementary Note 3.** $S_0^2$ is the amplitude reduction factor; CN is the coordination number; R is interatomic distance (the bond length between central atoms and surrounding coordination atoms); $\sigma^2$ is Debye-Waller factor (a measure of thermal and static disorder in absorber-scatter distances); $\Delta E_0$ is edge-energy shift (the difference between the zero kinetic energy value of the sample and that of the theoretical model). R factor is used to value the goodness of the fitting.

* This value was fixed during EXAFS fitting, based on the known structure of Ru foil. Error bounds that characterize the structural parameters obtained by EXAFS spectroscopy were estimated as N ± 20%; R ± 1%; $\sigma^2$ ± 20%; $\Delta E_0$ ± 20%.

**Supplementary Table 3.** Biomass compositional analysis of castor seed coats (endocarp) and isolated C-lignin (endocarp).

| Entry | Samples | Yield of lignin (wt%)[a] | Klason lignin (wt%) | Glucose (wt%) | Xylose (wt%) | Ash (wt%) |
|---|---|---|---|---|---|---|
| 1 | Castor seed coats | - | 59.3 | 15.6 | 9.5 | 3.1 |
| 2 | Isolated C-lignin | 21.0 | 83.1 | 0.6 | 2.1 | -- |

[a] Based on the content of Klason lignin in castor seed coats (endocarp).

**Supplementary Note 4.** The content of Klason lignin and carbohydrate in castor seed coats (endocarp) was measured according to NREL/TP-510-42618 protocol.[3] In a typical step: the ball milled castor seed coats (endocarp) or C-lignin (endocarp) (300 mg) and 3 mL of 72% (w/w) sulfuric acid were charged in a pressure tube. After stirring at 30 $^oC$ for 60 min, the mixture was diluted with deionized water (84 mL, the concentration of 72% is *ca*. 4%). The tube was sealed and placed into high pressure sterilizer at 121 $^oC$ for 60 min. When the reactor was cooled to ambient temperature, the solid and liquid were separated by filter crucible (filter diameter 4-7 um). The solid residue was dried at 110 $^oC$ to obtain Klason lignin. Subsequently, the mother liquid was diluted 50 times using the deionized water, filtered by 0.22 um syringe filter, and measured by HPAEC to obtain the contents of carbohydrates.

**Supplementary Note 5.** The ash in castor seed coats (endocarp) was analyzed according to NREL/TP-510-42622 protocol.[4] The dried sample (mass, M1) was placed into quartz tube and treated under 575 $^oC$ for 4 h under air atmosphere. After quartz tube cool to room temperature, the remaining solid was treated again under same conditions until the content of ash is constant weight (mass, M2). The ash content was obtained according to M2/M1 x 100%.

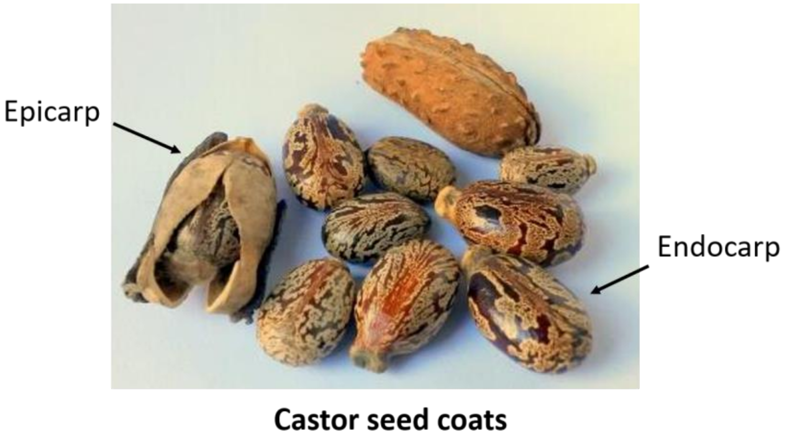


**Supplementary Figure 6.** The pictures of castor seed coats.

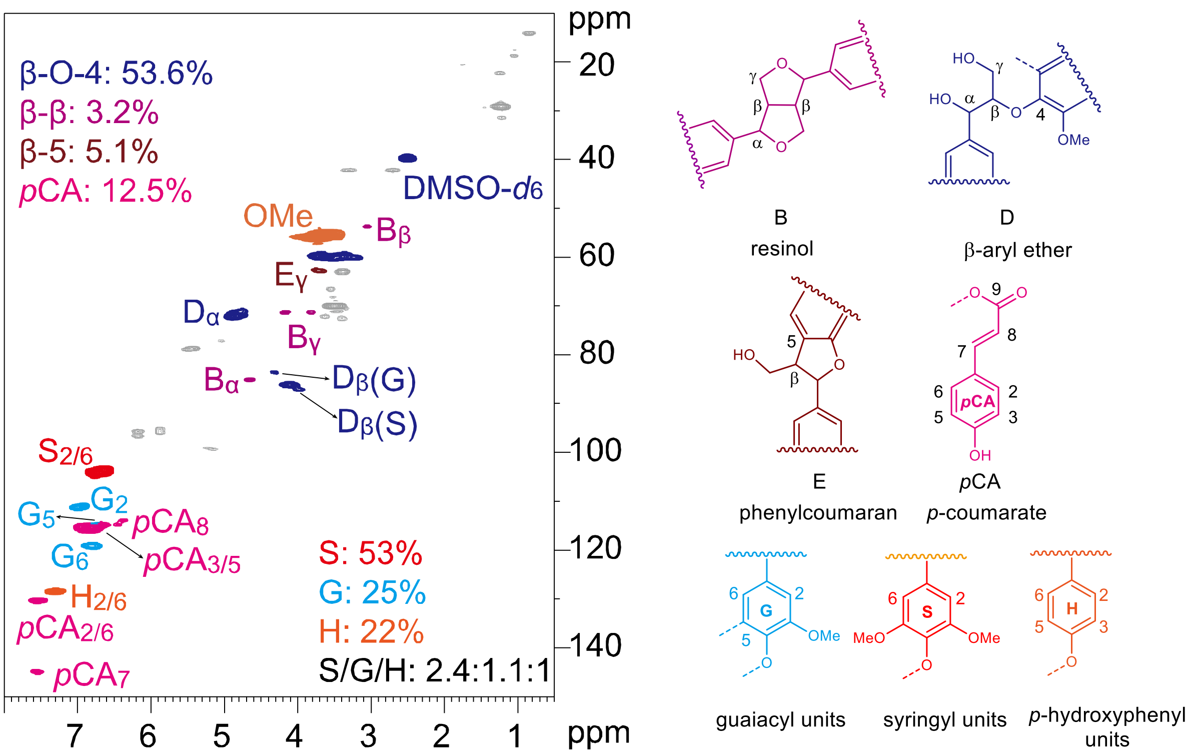


**Supplementary Figure 7.** 2D NMR spectrum of lignin isolated from castor seed coats (epicarp) (DMSO-$d_6$) and corresponding structures.

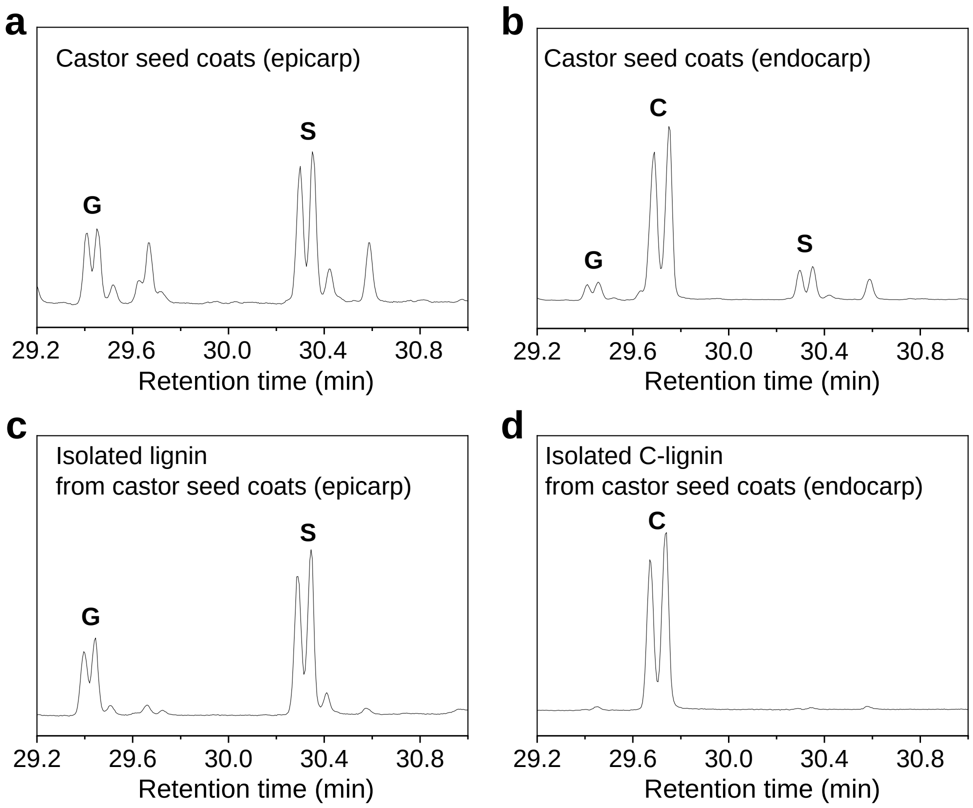


**Supplementary Figure 8.** GC-MS spectra from thioacidolysis analysis. **a** castor seed coats (epicarp). **b** castor seed coats (endocarp). **c** isolated lignin from castor seed coats (epicarp). **d** isolated C-lignin from castor seed coats (endocarp).

**Supplementary Note 6.** Lignin samples were isolated through the combination of enzymatic and mild acidolysis treatment (see **Methods**).

**Supplementary Discussion.** For raw material or isolated lignin from epicarp of castor seed coats, analysis of lignin monomer composition by thioacidolysis indicated that the lignin was composed of G and S units without any trace of C units (Supplementary Figs. 8a and 8c). In raw endocarp of castor seed coats, C units were the main ingredient with observation of G and S units (Supplementary Figure 8b). The G and S units were no longer observed in isolated C-lignin sample (Supplementary Figure 8d). It was proposed that the removal of G and S units occurred during mild acidolysis treatment process (to form a precipitate). Of note, the real C-lignin level in raw endocarp of castor seed coats should be high than the value estimated from the released thioacidolysis monomers, because C-lignin biopolymer comprised of benzodioxanes are substantially resistant to thioacidolysis treatments.[5,6]

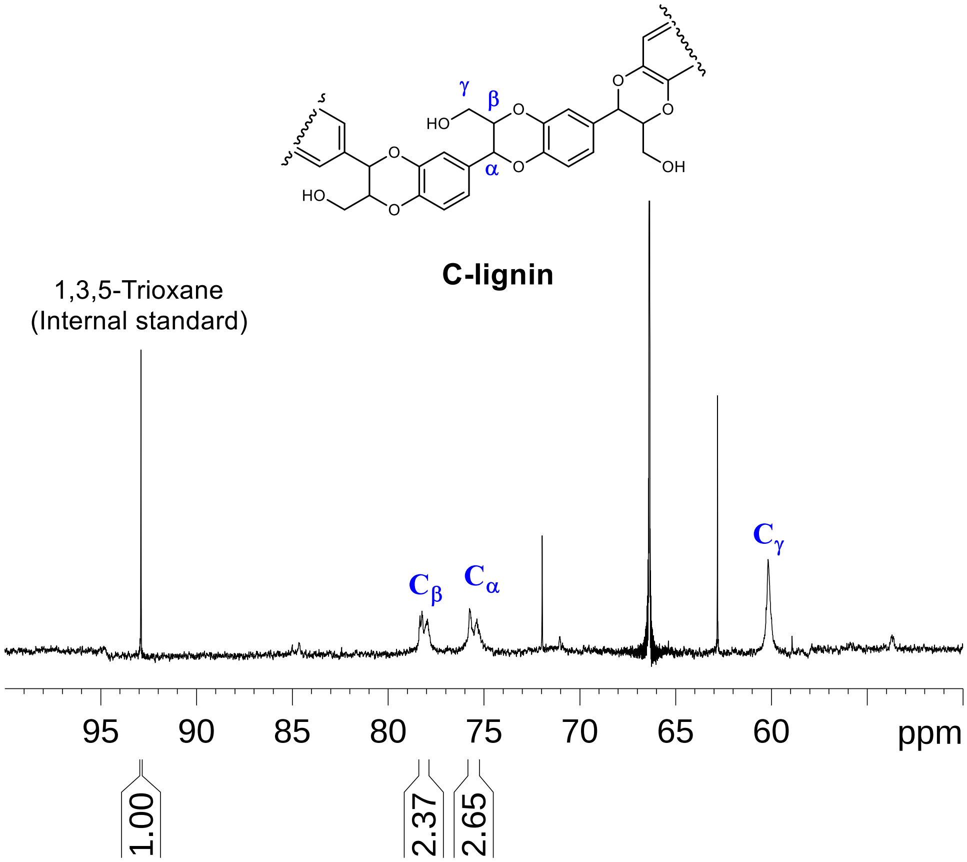


**Supplementary Figure 9.** Quantitative $^{13}$C NMR spectrum of isolated C-lignin (endocarp) (DMSO-$d_6$).

**Supplementary Note 7.** The quantification of caffeyl alcohol units in isolated C-lignin (endocarp) was performed according to previous report,[7] and $C_\beta$ was chosen as calculation refer.

**Supplementary Table 4.** Products distribution of catalytic hydrogenolysis of C-lignin (endocarp) with different catalysts.[a]

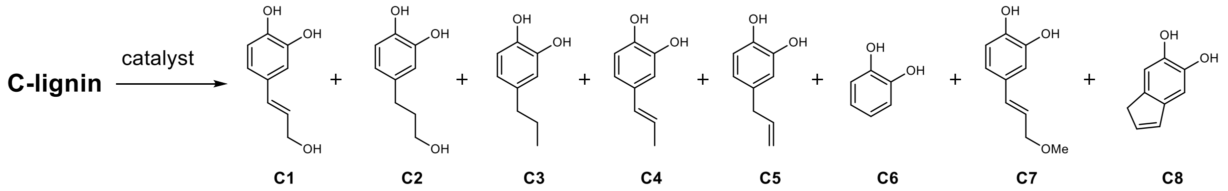


| Catalyst | Oily product (wt%) | Catechols yield (mol %)[b] | | | | | | | | | TON[c] | Selectivity (**C4**) (mol%)[d] | Solid recovery (wt%)[e] | Mass balance (wt%)[f] |
|---|---|---|---|---|---|---|---|---|---|---|---|---|---|---|
| | | **C1** | **C2** | **C3** | **C4** | **C5** | **C6** | **C7** | **C8** | Total | | | | |
| **no** | 40 | --[g] | -- | -- | -- | -- | -- | 4.3 | 5.5 | 9.8 | -- | 0 | 47 | 87 |
| **ZnO/C** | 30 | -- | -- | -- | 4 | 1 | 1 | -- | 5 | 11 | -- | 36 | 56 | 89 |
| **Ru/ZnO/C** | 70 | -- | 2.2 | 6.6 | 51 | 3.3 | 1.1 | -- | 2.2 | 66.4 | 278 | 77 | 18 | 91 |
| **Ru/C** | 66 | -- | 26.1 | 40.5 | 0.9 | -- | 0.9 | -- | -- | 68.4 | 11.4 | 1 | 25 | 93 |
| **Ru/C[h]** | 69 | -- | 20 | 70 | -- | -- | 2 | -- | -- | 92 | 9 | 0 | 23 | 95 |

[a] Reaction conditions: C-lignin (endocarp) (50 mg), catalyst (15 mg, 30 wt%) or no catalyst, MeOH (10 mL), 200 °C, 3 MPa $H_2$, and 4 h; [b] based on the molar amount of caffeyl alcohol in C-lignin; [c] The turnover numbers (TON) was calculated based on the total number of moles of Ru metal in the catalyst ($mol_{catechols}\ mol_{Ru}^{-1}$); [d] The mole ratios were determined by comparison of C-lignin hydrogenolysis products with authentic samples on GC; [e] Solid recovery contained catalyst and unreacted material (solid residue); [f] The mass of solid residue plus oily product; [g] -- represented the corresponding compounds were not detected; [h] 25 mg (50 wt%) Ru/C catalyst was used.

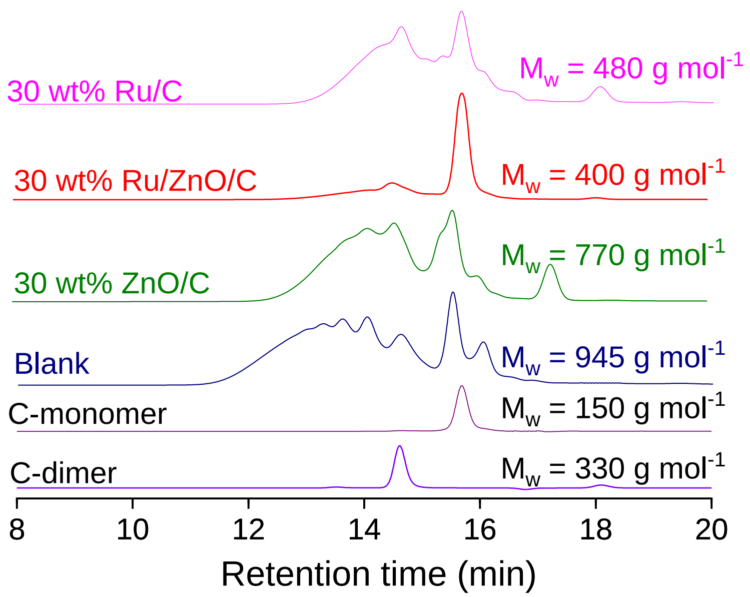


**Supplementary Figure 10.** The molecular weight distribution of oily products obtained from catalytic hydrogenolysis of C-lignin (endocarp) with different catalysts.

**Supplementary Table 5.** Elemental analysis of isolated C-lignin (endocarp) and oily product derived from Ru/ZnO/C-catalyzed hydrogenolysis of isolated C-lignin (endocarp).

| Entry | Samples | C (%) | H (%) | N (%) |
|---|---|---|---|---|
| 1 | Isolated C-lignin | 59.43 | 6.26 | 0.38 |
| 2 | Oily product[a] | 63.54 | 6.46 | 0.44 |

[a] Reaction conditions: C-lignin (endocarp) (50 mg), Ru/ZnO/C (15 mg, 30 wt%), MeOH (10 mL), 200 °C, 3 MPa $H_2$, and 4 h.

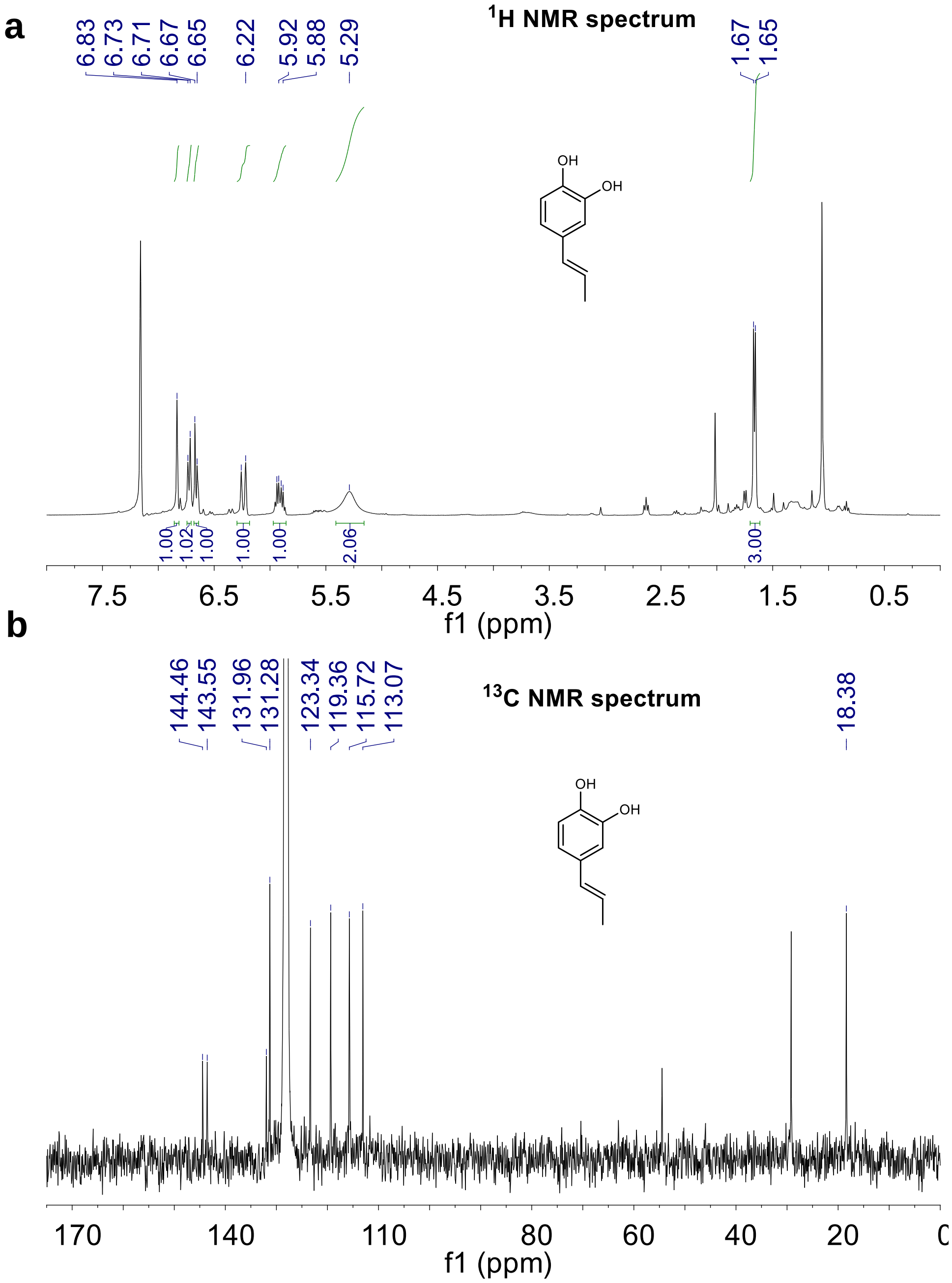


**Supplementary Figure 11.** NMR spectra of propenylcatechol **C4** isolated from hydrogenolysis of C-lignin (endocarp) ($C_6D_6$). **a** $^{1}$H NMR spectrum. **b** $^{13}$C NMR spectrum.

**Supplementary Note 8.** Reaction conditions: C-lignin (endocarp) (100 mg), Ru/ZnO/C (30 mg), MeOH (20 mL), 200 °C, $H_2$ (3 MPa), 4 h. The hydrogenolysis reaction gave an oily product after filtration and evaporation, which underwent silica gel column chromatography (EA/PE) to afford pure propenylcatechol **C4** (18 mg). NMR spectra of isolated propenylcatechol **C4** are constituent with the authentic sample.

**Supplementary Table 6.** Products distribution by using spent Ru/ZnO/C catalyst.[a]

| Run time | Oily product (wt%) | Catechols yield (mol %)[b] | | | | | | | | | TON[c] | Selectivity (C4) (mol %)[d] | Solid recovery (wt%)[e] | Mass balance (wt%)[f] |
|---|---|---|---|---|---|---|---|---|---|---|---|---|---|---|
| | | **C1** | **C2** | **C3** | **C4** | **C5** | **C6** | **C7** | **C8** | Total | | | | |
| 1 | 70 | --[g] | 2.2 | 6.6 | 51 | 3.3 | 1.1 | -- | 2.2 | 66.4 | 278 | 77 | 18 | 91 |
| 2 | 63 | -- | 2.2 | 9.9 | 43.8 | 1.1 | 2.2 | -- | 1.1 | 60.3 | 253 | 73 | 25 | 92 |
| 3 | 62 | -- | 2.2 | 9.9 | 44 | 2.2 | 2.2 | -- | 1.1 | 61.6 | 259 | 71 | 27 | 94 |
| 4 | 65 | -- | 2.2 | 7.7 | 47.3 | 2.2 | 2.2 | -- | 2.2 | 63.8 | 265 | 74 | 21 | 93 |
| 5 | 62 | -- | 2.2 | 6.6 | 42.9 | 2.2 | 2.2 | -- | 2.2 | 58.3 | 245 | 73.5 | 17 | 90 |
| 6 | 61 | -- | 2.2 | 4.4 | 41.8 | 2.2 | 2.2 | -- | 3.3 | 56.1 | 230 | 74.5 | 14 | 89 |

[a] Reaction conditions: C-lignin (endocarp) (50 mg), Ru/ZnO/C (15 mg, 30 wt%), MeOH (10 mL), 200 °C, 3 MPa $H_2$, and 4 h; [b] based on the molar amount of caffeyl alcohol in C-lignin; [c] The turnover numbers (TON) was calculated based on the total number of moles of Ru metal in the catalyst ($mol_{catechols}$ $mol_{Ru}^{-1}$); [d] The mole ratios were determined by comparison of C-lignin hydrogenolysis products with authentic samples on GC; [e] Solid recovery contained catalyst and unreacted material (solid residue); [f] The mass of solid residue plus oily product; [g] -- represented the corresponding compounds were not detected.

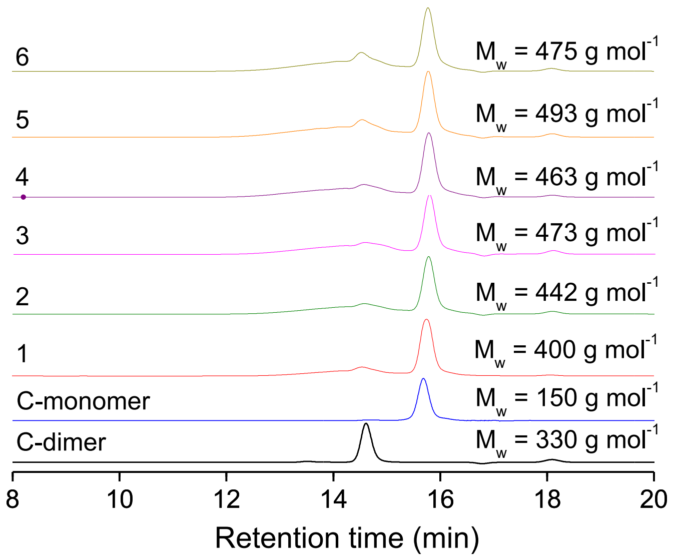


**Supplementary Figure 12.** The molecular weight distribution of C-lignin (endocarp) oily product by using spent Ru/ZnO/C catalyst.

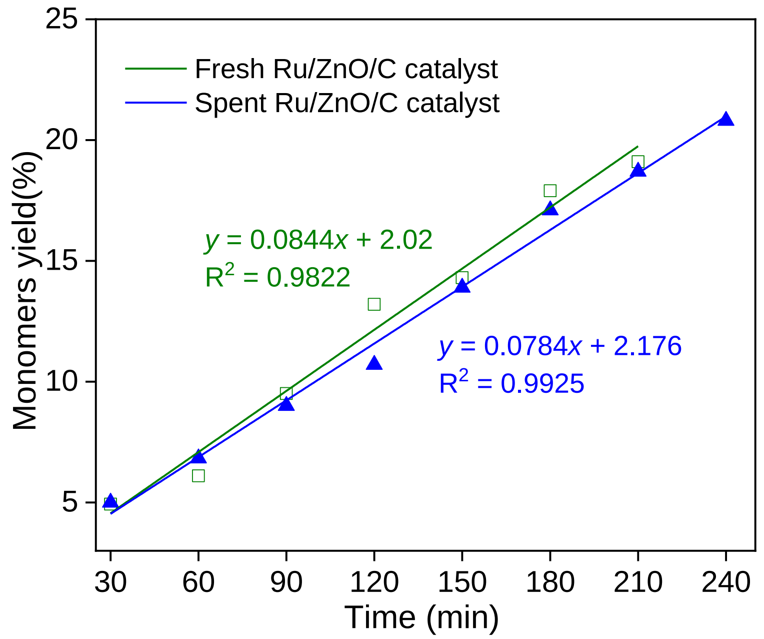


**Supplementary Figure 13.** Kinetic data of C-lignin (endocarp) hydrogenolysis by fresh and spent Ru/ZnO/C catalysts.

**Supplementary Note 9.** Kinetic experiments were performed in a 300 mL Parr autoclave equipped with a sampling device and a constant pressure complementary $H_2$ device. The reaction temperature was set as 180 ºC. *For fresh catalyst*, C-lignin (endocarp) (180 mg), Ru/ZnO/C (36 mg, 20 wt%), 1,3,5-trimethoxybenzene (20 mg, internal standard) and MeOH (150 mL) were mixed in the autoclave, which was then exchanged and pressured with $H_2$ (400 psi at room temperature). The reaction temperature was raised to 180 ºC, and the pressure was boosted to 720 psi. An aliquot (*ca.* 2 mL) was taken every 30 minutes, which underwent the removal of methanol under vacuum and the treatment with BSTFA (150 μL) in anhydrous THF (1 mL) before GC analysis. The *spent catalyst* was recovered by centrifugation, being washed with methanol and dried under vacuum, which was then used in the following cycle under same conditions.

Comparable kinetic data were obtained from fresh and spent Ru/ZnO/C catalysts. In view of that it is difficult to recover catalyst completely under such a scale, we thought there is no obvious loss of activity in the spent Ru/ZnO/C catalyst.

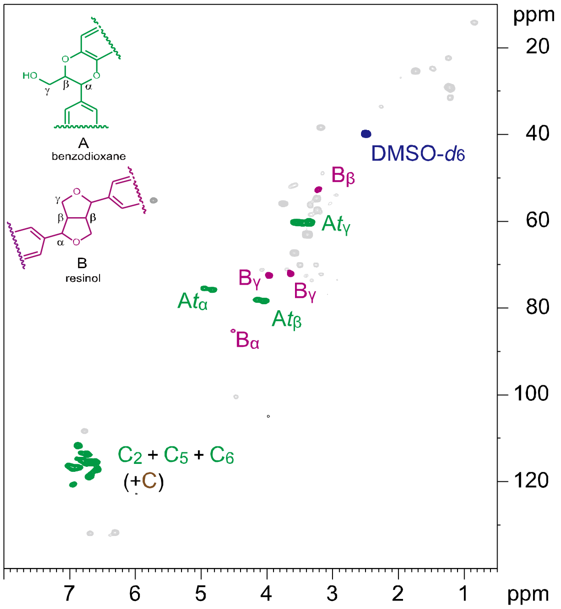


**Supplementary Figure 14.** 2D NMR spectrum of C-lignin (endocarp) oily product derived from catalyst-free condition (DMSO-$d_6$).

**Supplementary Table 7.** Products distribution of Ru/ZnO/C-catalyzed hydrogenolysis of C-lignin (endocarp) in different solvents.[a]

| Solvent | Oily product (wt%) | Catechols yield (mol %)[b] | | | | | | | | | TON[c] | Selectivity (**C4**) (mol %)[d] | Solid recovery (wt%)[e] | Mass balance (wt%)[f] |
|---|---|---|---|---|---|---|---|---|---|---|---|---|---|---|
| | | **C1** | **C2** | **C3** | **C4** | **C5** | **C6** | **C7** | **C8** | Total | | | | |
| MeOH | 70 | --[g] | 2.2 | 6.6 | 51 | 3.3 | 1.1 | -- | 2.2 | 66.4 | 278 | 77 | 18 | 91 |
| EtOH | 64 | -- | 2 | 7 | 40 | 4 | 1 | -- | 1 | 55 | 231 | 73 | 27 | 93 |
| $^{i}$PrOH | 53 | 4 | 2 | 1 | 22 | 3 | 1 | -- | 2 | 35 | 147 | 63 | 35 | 90 |
| dioxane | 89 | 3 | -- | 1 | 9 | 2 | -- | -- | 1 | 16 | 67 | 56 | 7 | 97 |
| THF | 69 | 4 | -- | 1 | 8 | 1 | -- | -- | 1 | 15 | 63 | 53 | 25 | 95 |

[a] Reaction conditions: C-lignin (endocarp) (50 mg), Ru/ZnO/C (15 mg, 30 wt%), solvent (10 mL), 200 °C, 3 MPa $H_2$, and 4 h; [b] based on the molar amount of caffeyl alcohol in C-lignin; [c] The turnover numbers (TON) was calculated based on the total number of moles of Ru metal in the catalyst ($mol_{catechols}\ mol_{Ru}^{-1}$); [d] The mole ratios were determined by comparison of C-lignin hydrogenolysis products with authentic samples on GC; [e] Solid recovery contained catalyst and unreacted material (solid residue); [f] The mass of solid residue plus oily product; [g] -- represented the corresponding compounds were not detected.

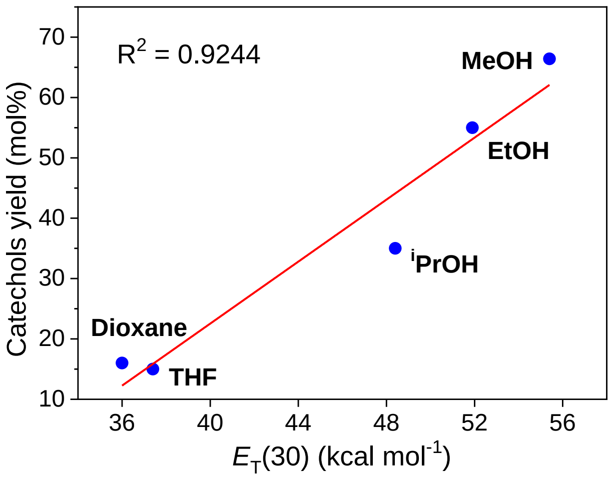


**Supplementary Figure 15.** The relationship between catechols yields and solvent polarity ($E_T(30)$).

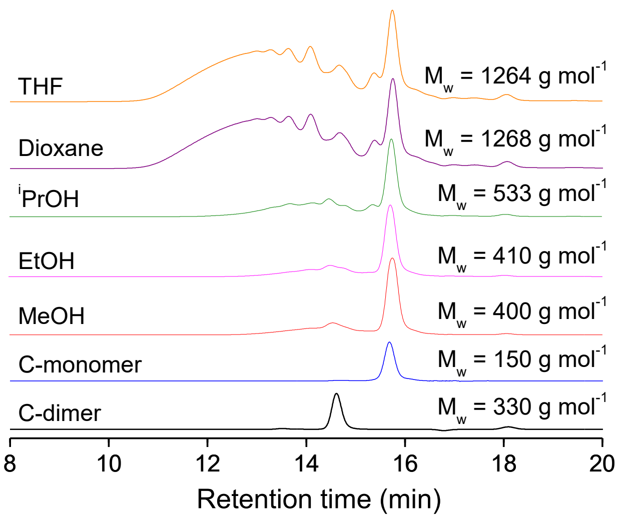


**Supplementary Figure 16.** The molecular weight distribution of C-lignin (endocarp) oily product in different solvents.

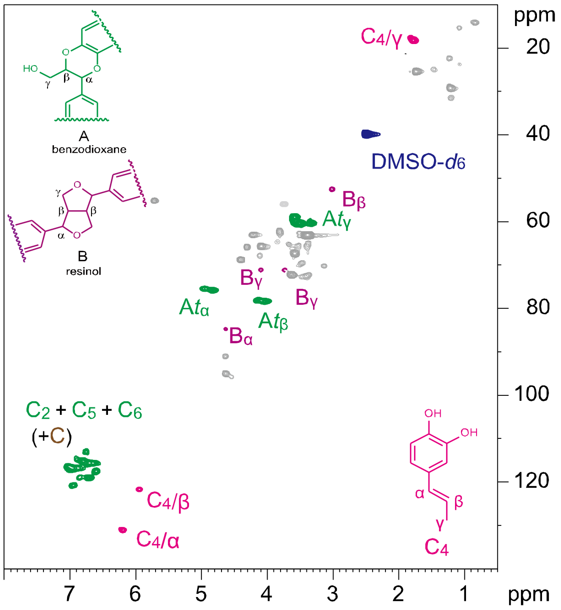


**Supplementary Figure 17.** 2D NMR spectrum of C-lignin (endocarp) oily product from hydrogenolysis in dioxane (DMSO-*d*6).

**Supplementary Table 8.** Products distribution of Ru/ZnO/C-catalyzed hydrogenolysis of C-lignin (endocarp) at different reaction temperatures.[a]

| Temp. (°C) | Oily product (wt%) | Catechols yield (mol %)[b] | | | | | | | | | TON[c] | Selectivity (mol %)[d] | | Solid recovery (wt%)[e] | Mass balance (wt%)[f] |
|---|---|---|---|---|---|---|---|---|---|---|---|---|---|---|---|
| | | **C1** | **C2** | **C3** | **C4** | **C5** | **C6** | **C7** | **C8** | Total | | **C4** | **C3** | | |
| 180 | 73 | --[g] | 2 | 4 | 30 | 1 | 1 | -- | 2 | 40 | 168 | 75 | 10 | 21 | 95 |
| 200 | 70 | -- | 2.2 | 6.6 | 51 | 3.3 | 1.1 | -- | 2.2 | 66.4 | 278 | 77 | 10 | 18 | 91 |
| 220 | 57 | -- | 4 | 42 | 27 | -- | 3 | -- | -- | 76 | 319 | 36 | 55 | 23 | 88 |
| 240 | 61 | -- | 9 | 59 | 12 | -- | 2 | -- | -- | 82 | 345 | 15 | 72 | 20 | 85 |

[a] Reaction conditions: C-lignin (endocarp) (50 mg), Ru/ZnO/C (15 mg, 30 wt%), MeOH (10 mL), 3 MPa $H_2$, and 4 h; [b] based on the molar amount of caffeyl alcohol in C-lignin; [c] The turnover numbers (TON) was calculated based on the total number of moles of Ru metal in the catalyst ($mol_{catechols}$ $mol_{Ru}^{-1}$); [d] The mole ratios were determined by comparison of C-lignin hydrogenolysis products with authentic samples on GC; [e] Solid recovery contained catalyst and unreacted material (solid residue); [f] The mass of solid residue plus oily product; [g] -- represented the corresponding compounds were not detected.

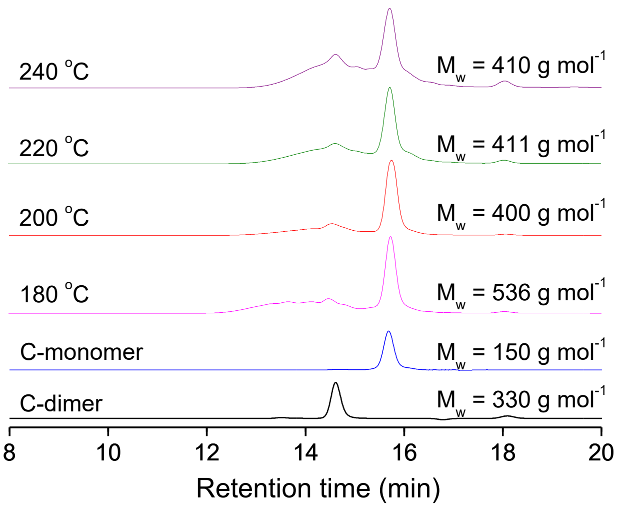


**Supplementary Figure 18.** The molecular weight distribution of C-lignin (endocarp) oily product obtained at different reaction temperatures.

**Supplementary Table 9.** Products distribution of Ru/ZnO/C-catalyzed hydrogenolysis of C-lignin (endocarp) with different reaction time.[a]

| Time (h) | Oily product (wt%) | Catechols yield (mol %)[b] | | | | | | | | | TON[c] | Selectivity (mol %)[d] | | Solid recovery (wt%)[e] | Mass balance (wt%)[f] |
|---|---|---|---|---|---|---|---|---|---|---|---|---|---|---|---|
| | | **C1** | **C2** | **C3** | **C4** | **C5** | **C6** | **C7** | **C8** | Total | | **C4** | **C3** | | |
| 2 | 57 | --[g] | 1 | 1 | 28 | 4 | 1 | -- | 5 | 40 | 168 | 70 | 3 | 32 | 91 |
| 4 | 70 | -- | 2.2 | 6.6 | 51 | 3.3 | 1.13 | -- | 2.2 | 66.4 | 278 | 77 | 10 | 18 | 91 |
| 6 | 68 | -- | 4 | 45 | 22 | -- | 2 | -- | -- | 73 | 306 | 30 | 61 | 18 | 89 |
| 12 | 60 | -- | 6 | 47 | 21 | -- | 2 | -- | -- | 76 | 319 | 27 | 62 | 20 | 85 |

[a] Reaction conditions: C-lignin (endocarp) (50 mg), Ru/ZnO/C (15 mg, 30 wt%), MeOH (10 mL), 200 °C, 3 MPa $H_2$; [b] based on the molar amount of caffeyl alcohol in C-lignin; [c] The turnover numbers (TON) was calculated based on the total number of moles of Ru metal in the catalyst ($mol_{catechols}$ $mol_{Ru}^{-1}$); [d] The mole ratios were determined by comparison of C-lignin hydrogenolysis products with authentic samples on GC; [e] Solid recovery contained catalyst and unreacted material (solid residue); [f] The mass of solid residue plus oily product; [g] -- represented the corresponding compounds were not detected.

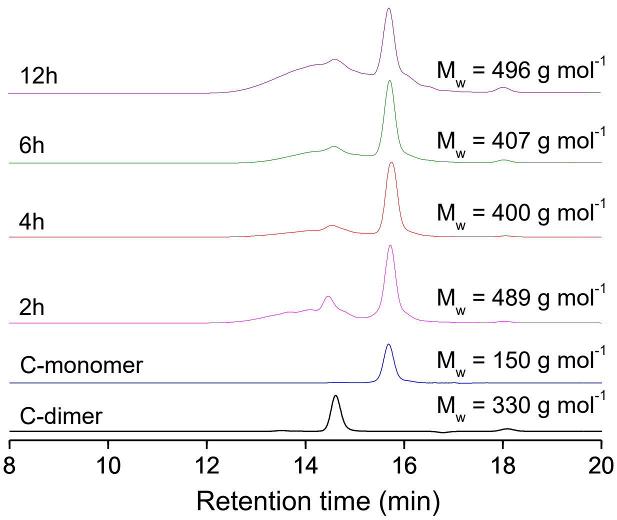


**Supplementary Figure 19.** The molecular weight distribution of C-lignin (endocarp) oily product obtained at different reaction time.

**Supplementary Table 10.** Products distribution of Ru/ZnO/C-catalyzed hydrogenolysis of C-lignin (endocarp) with different catalyst dosage.[a]

| Catalyst dosage (wt%)[b] | Oily product (wt%) | Catechol yield (mol %)[c] | | | | | | | | | TON[d] | Selectivity (mol %)[e] | | Solid recovery (wt%)[f] | Mass balance (wt%)[g] |
|---|---|---|---|---|---|---|---|---|---|---|---|---|---|---|---|
| | | **C1** | **C2** | **C3** | **C4** | **C5** | **C6** | **C7** | **C8** | Total | | **C4** | **C3** | | |
| 10 | 52 | --[h] | -- | 2 | 15 | 2 | 1 | -- | 17 | 37 | 465 | 41 | 5 | 24 | 86 |
| 20 | 65 | -- | 2 | 4 | 41 | 4 | 2 | -- | 3 | 56 | 352 | 73 | 7 | 23 | 89 |
| 30 | 70 | -- | 2.2 | 6.6 | 51 | 3.3 | 1.1 | -- | 2.2 | 66.4 | 278 | 77 | 10 | 18 | 91 |
| 50 | 65 | -- | 3 | 31 | 31 | -- | 2 | -- | -- | 67 | 169 | 46 | 46 | 24 | 93 |
| 100 | 90 | -- | 7 | 60 | 10 | -- | 2 | -- | -- | 79 | 100 | 13 | 76 | 2 | 96 |

[a] Reaction conditions: C-lignin (endocarp) (50 mg), Ru/ZnO/C (15 mg, 30 wt%), MeOH (10 mL), 200 °C, 3 MPa $H_2$; [b] the percentage of catalyst dosage based on C-lignin mass; [c] based on the molar amount of caffeyl alcohol in C-lignin; [d] The turnover numbers (TON) was calculated based on the total number of moles of Ru metal in the catalyst ($mol_{catechols}\ mol_{Ru}^{-1}$); [e] The mole ratios were determined by comparison of C-lignin hydrogenolysis products with authentic samples on GC; [f] Solid recovery contained catalyst and unreacted material (solid residue); [g] The mass of solid residue plus oily product; [h] -- represented the corresponding compounds were not detected.

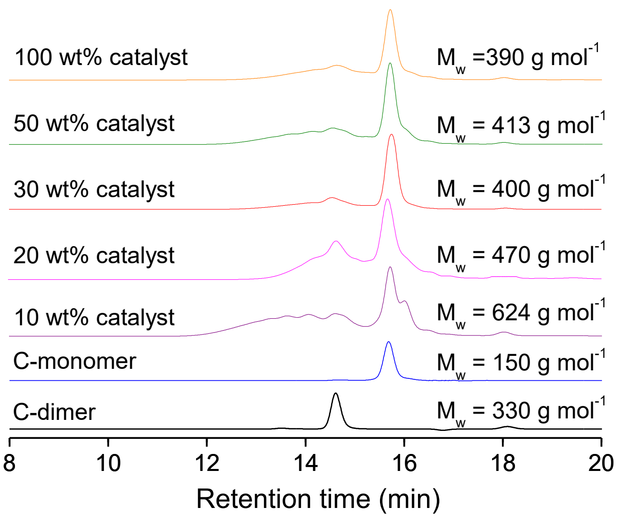


**Supplementary Figure 20.** The Molecular weight distribution of C-lignin (endocarp) oily product obtained with different catalyst dosage.

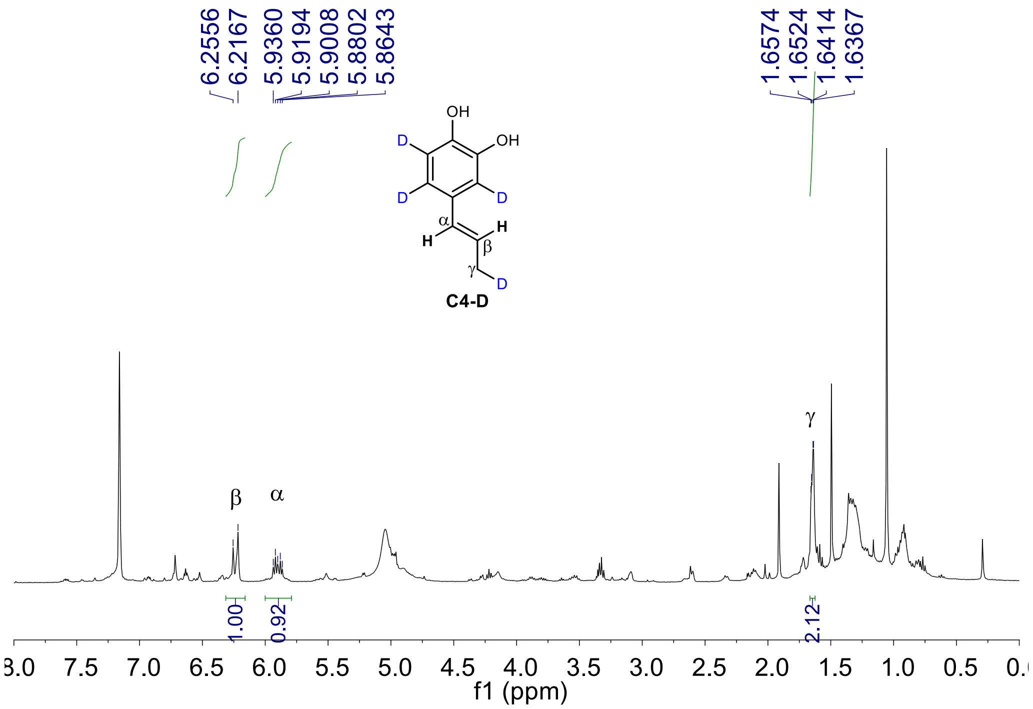


**Supplementary Figure 21.** $^1H$ NMR spectrum of propenylcatechol **C4-D** isolated from hydrogenolysis of **M1** in a deuterated condition ($C_6D_6$).

**Supplementary Note 10.** Dimeric compound **M1** (50 mg), Ru/ZnO/C (10 mg, 20 wt%) and $CD_3OD$ (10 mL) were charged in a 50 mL Parr autoclave, which was purged with nitrogen and pressured to $D_2$ (1 MPa) at room temperature. After reaction, the oily product was obtained by filtration and evaporation. The analysis by GC-MS after trimethylsilylation indicated that propenylcatechol **C4-D** was generated as major product (65%), together with observation of **C3**, **C2** and **C5**. A mass value for silylated **C4-D** was measured as 298.2 g $mol^{-1}$, suggesting four protons have been deuterated.

**C4-D** could be isolated from the oily product by silica gel column chromatography (EA/PE). $^1H$ NMR spectroscopic analysis indicated that the protons at aromatic ring has been exchanged by deuterium, being consistent with the observation in mass spectrum. The signals resonated at 6.23 ppm (d, $J$ = 15.6 Hz, 1 H), 5.86-5.94 ppm (m, 1 H), and 1.64-1.66 ppm (m, 2 H) still remained.

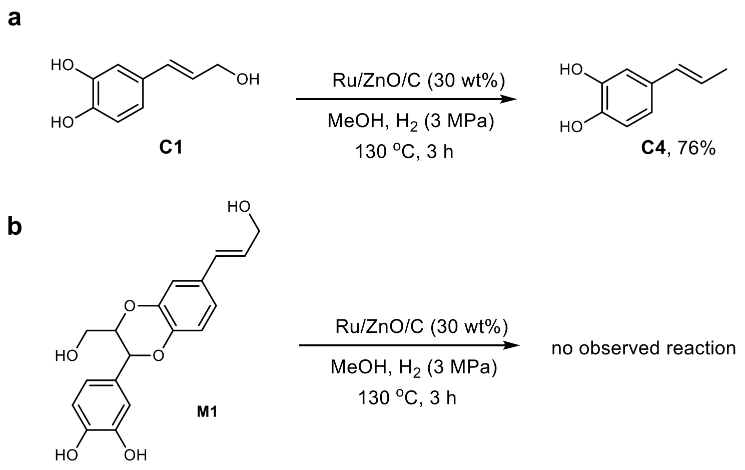


**Supplementary Figure 22.** Ru/ZnO/C-catalytic treatment model compounds at low reaction temperature. **a** Catalytic treatment of **C1**. **b** Catalytic treatment of **M1**.

**Supplementary Note 11.** These two reactions were performed by using **C1** or **M1** (50 mg) with Ru/ZnO/C (15 mg) under $H_2$ (2 MPa) at **130 °C** for 3 h. After filtration, MeOH was removed by rotary evaporator and the residue was dissolved in DMSO-$d_6$. $^1$H NMR spectra of crude products indicated that caffeyl alcohol **C1** underwent hydrogenolysis of allylic alcohol to give **C4** in 76% yield, while **M1** remained nearly intact.

**Supplementary Table 11.** NMR data for the signal assignments in C-lignin (endocarp) sample and lignin oily products (endocarp) in DMSO-$d_6$.[8]

| Lable | $\delta_C/\delta_H$ (ppm) | Assignment |
|---|---|---|
| A$t_\alpha$ | 75.4/4.83 | $C_\alpha$-$H_\alpha$ in *trans*-benzodioxane substructures (A) |
| A$t_\beta$ | 77.9-4.06 | $C_\beta$-$H_\beta$ in *trans*-benzodioxane substructures (A) |
| A$t_\gamma$ | 59.9/3.34-3.55 | $C_\gamma$-$H_\gamma$ in *trans*-benzodioxane substructures (A) |
| A$c_\alpha$ | 74.8-5.18 | $C_\alpha$-$H_\alpha$ in *cis*-benzodioxane substructures (A) |
| A$c_\beta$ | 76.9-4.38 | $C_\beta$-$H_\beta$ in *cis*-benzodioxane substructures (A) |
| A$c_\beta$ | 58.4-3.35 | $C_\gamma$-$H_\gamma$ in *cis*-benzodioxane substructures (A) |
| $B_\alpha$ | 84.5/4.62 | $C_\alpha$-$H_\alpha$ in β-β resinol substructures (B) |
| $B_\beta$ | 53.4/3.04 | $C_\beta$-$H_\beta$ in β-β resinol substructures (B) |
| $B_\gamma$ | 70.7/3.74-4.09 | $C_\gamma$-$H_\gamma$ in β-β resinol substructures (B) |
| $C_\alpha$ | 127.9/6.42 | $C_\alpha$-$H_\alpha$ in cinnamyl alcohol end-units (C) |
| $C_\beta$ | 128.7/6.20 | $C_\beta$-$H_\beta$ in cinnamyl alcohol end-units (C) |
| $C_\gamma$ | 61.4/4.10 | $C_\gamma$-$H_\gamma$ in cinnamyl alcohol end-units (C) |
| $C_2$, $C_5$, $C_6$ | 115.8/6.77, 116.5/6.95, 118.6/6.70, 120.4/6.95, | $C_2$-$H_2$, $C_5$-$H_5$, $C_6$-$H_6$ in catechgylunits (C) |
| **C3** (α) | 37.2/3.24 | $C_\alpha$-$H_\alpha$ in **C3** |
| **C3** (β) | 24.5/1.50 | $C_\beta$-$H_\beta$ in **C3** |
| **C3** (γ) | 13.9/0.83 | $C_\gamma$-$H_\gamma$ in **C3** |
| **C4** (α) | 130.8/6.22 | $C_\alpha$-$H_\alpha$ in **C4** |
| **C4** (β) | 123.1/5.90 | $C_\beta$-$H_\beta$ in **C4** |
| **C4** (γ) | 17.9/1.66 | $C_\gamma$-$H_\gamma$ in **C4** |

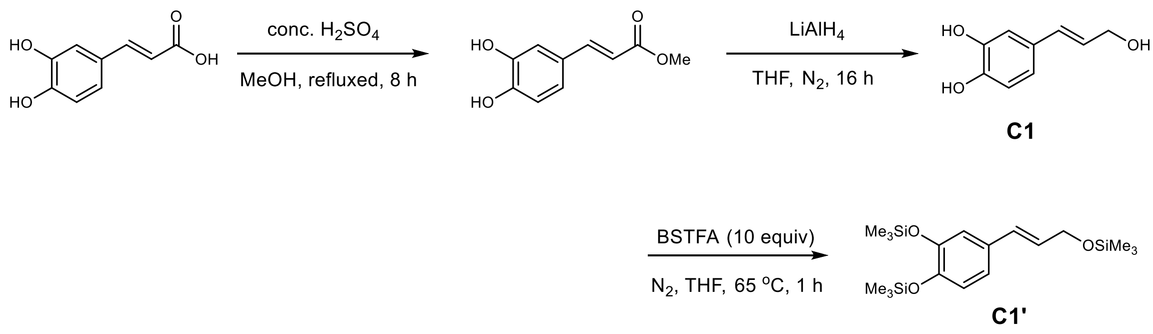


**Supplementary Figure 23.** The synthesis of caffeyl alcohol **C1** and silylated derivative.

**Supplementary Method.** The **C1** synthesis steps was coincident with our previous reported literature.[8] The acquired caffeyl alcohol **C1** (100 mg, 0.6 mmol) was treated with excess *N*,*O*-bis(trimethylsilyl)trifluoroacetamide (1.55 g, 6 mmol) in anhydrous THF (10 mL) at 65 $^{o}$C for 1 h under $N_2$. The silylated product **C1’** was purified by column chromatography (PE) (206 mg, 90%). **$^{1}$H NMR** (400 MHz, $CDCl_3$): δ 6.88-6.85 (m, 2 H), 6.76 (d, *J* = 8.0 Hz, 1 H), 6.45 (d, *J* = 15.8 Hz, 1 H), 6.15-6.08 (m, 1 H), 4.28 (dd, *J* = 1.2 Hz, 5.6 Hz, 2 H), 0.25 (d, *J* = 2.6 Hz, 18 H), 0.16 (s, 9 H). **$^{13}$C NMR** (100 MHz, $CDCl_3$): 146.6, 146.4, 131.3, 130.1, 127.0, 121.0, 120.4, 119.0, 63.7, 0.48, 0.14.

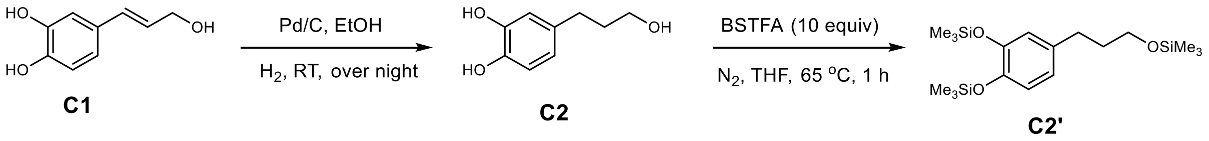


**Supplementary Figure 24.** The synthesis of **C2** and silylated derivative.[8]

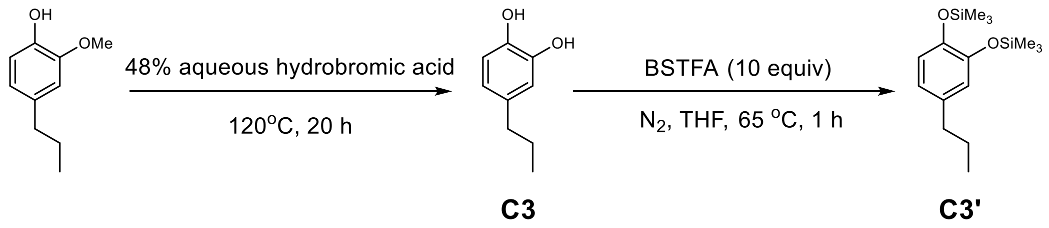


**Supplementary Figure 25.** The synthesis of **C3** and silylated derivative.[8]

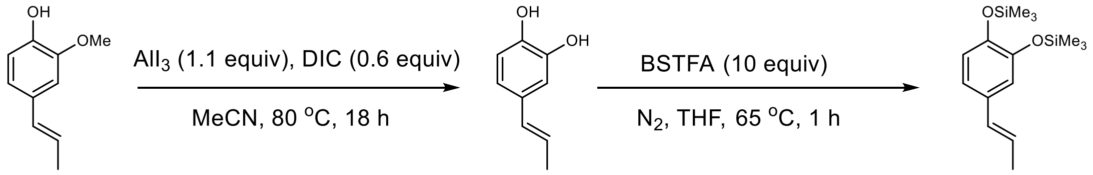


**C4** **C4'**

**Supplementary Figure 26.** The synthesis of **C4** and silylated derivative.

**Supplementary Method.** 1,3-Diisopropylcarbodiimide (DIC) (46.1 mg, 0.37 mmol, 0.6 equiv) and isoeugenol (100 mg, 0.61 mmol) were added sequentially to a suspension of $AlI_3$ (273 mg, 0.67 mmol, 1.1 equiv) in $CH_3CN$ (15 mL) under $N_2$ atmosphere. The mixture was stirred at 80 °C for 18 h, which was acidified with HCl and extracted with EtOAc. The organic phase was washed with saturated $Na_2S_2O_3$ solution and brine, and was dried with anhydrous $Na_2SO_4$. The solvent was removed via rotary evaporator and the residue was purified by column chromatography (PE/EtOAc, 4:1) to yield **C4** as a pale yellow solid (84 mg, 92%). **$^1$H NMR** (400 MHz, $C_6D_6$): δ 6.73 (d, *J* = 2.0 Hz, 1 H), 6.70 (dd, *J* = 8.0 Hz, 2.0 Hz, 1 H), 6.57 (d, *J* = 8.0 Hz, 1 H), 6.22 (dd, *J* = 15.8 Hz, 1.6 Hz, 1 H), 5.94-5.85 (m, 1 H), 4.67 (s, 2 H), 1.66 (dd, *J* = 6.6 Hz, 1.6 Hz, 3 H). **$^{13}$C NMR** (100 MHz, $C_6D_6$):143.8, 142.9, 131.6, 130.8, 123.1, 119.0, 115.2, 112.6, 17.9.

**C4’** was prepared through a similar method to **C1’** by using **C4** as a starting material. (yield, 92%). **$^1$H NMR** (400 MHz, $CDCl_3$): δ 6.84-6.81 (m, 2 H), 6.75 (d, *J* = 8.0 Hz, 1 H), 6.28 (dd, *J* = 15.8 Hz, 1.6 Hz, 1 H), 6.11-6.02 (m, 1 H), 1.86-1.84 (dd, *J* = 6.6 Hz, 1.6 Hz, 3 H), 0.26 (d, *J* = 4.5 Hz, 18 H). **$^{13}$C NMR** (100 MHz, $CDCl_6$): 146.5, 145.6, 132.1, 130.5, 123.7, 120.9, 119.5, 18.3, 0.34,0.31.

The NMR spectra coincided with precious report in literatures.[9]

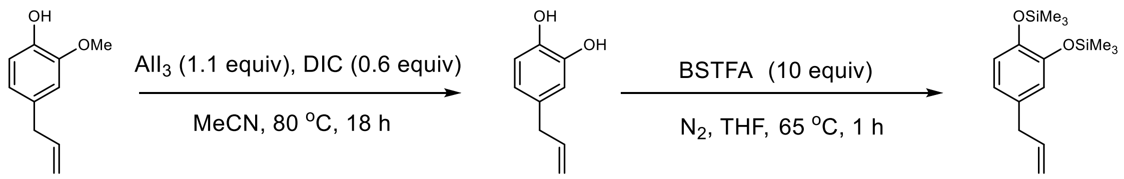


**C5** **C5'**

**Supplementary Figure 27.** The synthesis of **C5** and silylated derivative.

**Supplementary Method. C5** was prepared through a similar method to **C4** by using 4-allyl-2-methoxyphenol as a starting material. (yield, 92%). **$^1$H NMR** (400 MHz, $CDCl_3$): δ 6.79 (d, *J* = 8.0 Hz, 1 H), 6.71 (d, *J* = 2.0 Hz, 1 H), 6.63 (dd, *J* = 8.0 Hz, 2.0 Hz, 1 H), 5.97-5.87 (m, 1 H), 5.08-5.05 (m, 1 H), 5.04-5.02 (m, 1 H), 3.27 (d, *J* = 6.6 Hz, 2 H). **$^{13}$C NMR** (100 MHz, $CDCl_3$): δ 143.5, 141.7, 137.7, 133.4, 121.2, 115.8, 115.7, 115.5, 39.6.

**C5'** was prepared through a similar method to **C1'** by using **C5** as a starting material. (yield, 95%). **$^1$H NMR** (400 MHz, $CDCl_3$): δ 6.75 (d, *J* = 7.6 Hz, 1 H), 6.67-6.64 (m, 2 H), 6.01-5.90 (m, 1 H), 5.08-5.06 (m, 1 H), 5.03 (s, 1 H), 3.28 (d, *J* = 6.8 Hz, 2 H), 0.25 (s, 18 H). **$^{13}$C NMR** (100 MHz, $CDCl_3$): 146.3, 144.7, 137.7, 133.6, 121.7, 121.4, 120.7, 115.4, 39.5, 0.33.

The NMR spectra coincided with precious report in literatures.[9]

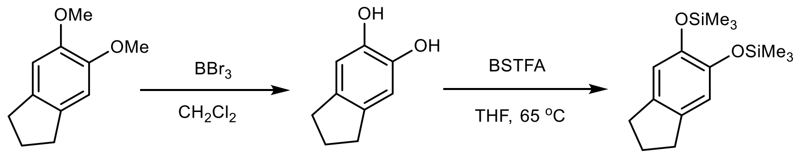


**Supplementary Figure 28.** The synthesis of 2,3-dihydro-1H-indene-5,6-diol and silylated derivative.

**Supplementary Method.** To a solution of 5,6-Dimethoxy-2,3-dihydro-1*H*-indene (500 mg, 2.8 mmol) in $CH_2Cl_2$ (5 mL) was added $BBr_3$ (0.63 mL, 2.4 equiv.) at 0 °C, and the mixture was allowed to stir at room temperature for 2 h. Water was added dropwise to quench the reaction, and the mixture was extracted with $CH_2Cl_2$. The organic phase was dried by $MgSO_4$ and evaporated under vacuum. The residue was purified by column chromatography (PE/EtOAc, 4:1) to yield 2,3-dihydro-1H-indene-5,6-diol as a white solid (350 mg, 84%). **$^1$H NMR** (400 MHz, $CDCl_3$): δ 6.75 (s, 2H), 4.74 (br, 2H), 2.80 (t, *J* = 7.4 Hz, 4H), 2.09-2.02 (m, 2H). **$^{13}$C NMR** (100 MHz, $CDCl_3$): δ 141.9, 136.5, 111.3, 32.5, 25.8.

The silylation of 2,3-dihydro-1H-indene-5,6-diol was conducted through above mentioned method, and 5,6-bis((trimethylsilyl)oxy)-2,3-dihydro-1H-indene was obtained as a colorless oil in 94% yield. **$^1$H NMR** (400 MHz, $CDCl_3$): δ 6.70 (s, 2H), 2.81 (t, *J* = 7.4 Hz, 4H), 2.06 (dq, *J* = 14.7, 7.4 Hz, 2H), 0.25 (s, 18H). **$^{13}$C NMR** (100 MHz, $CDCl_3$): δ 144.7, 137.0, 116.6, 32.6, 25.8, 0.35.

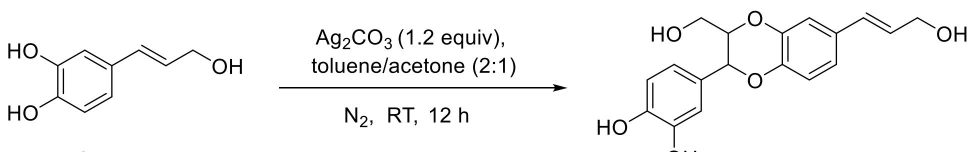


**Supplementary Figure 29.** The synthesis of dimetric model **M1**.

**Supplementary Method.** To a solution of caffeyl alcohol **C1** (1.0 g, 6.02 mmol) in anhydrous acetone-toluene (15 mL, 1:2, v/v) was added silver carbonate (2.0 g, 7.22 mmol, 1.2 equiv), and the mixture was stirred at RT for 12 h. After filtration, the liquid phase was concentrated and purified by column chromatography (PE/EtOAc, 1:1) to yield **M1** as a white solid (500 mg, 50%). **$^1$H NMR** (400 MHz, DMSO-$d_6$): δ 3.33-3.36 (m, 1 H), 3.52-3.54 (m, 1 H), 3.99-4.04 (m, 1 H), 4.07-4.08 (m, 2 H), 4.82 (d, $J$ = 7.6 Hz, 1 H), 6.17-6.23 (m, 1 H), 6.43 (d, $J$ = 16.0 Hz, 1 H), 6.70 (d, $J$ = 7.4 Hz, 1 H), 6.76 (d, $J$ = 8.0 Hz, 1 H), 6.82 (s, 1 H), 6.87 (d, $J$ = 8.0 Hz, 1 H), 6.90-6.93 (m, 1 H), 6.96 (s, 1H), 9.03 (s, 2 H). **$^{13}$C NMR** (100 MHz, DMSO-$d_6$): 145.8, 145.3, 143.7, 142.7, 130.4, 128.9, 128.2, 127.7, 119.5, 118.9, 116.9, 115.6, 115.0, 114.3, 78.4, 75.7, 61.7, 60.3. The NMR spectra coincided with precious report in literatures.[7]

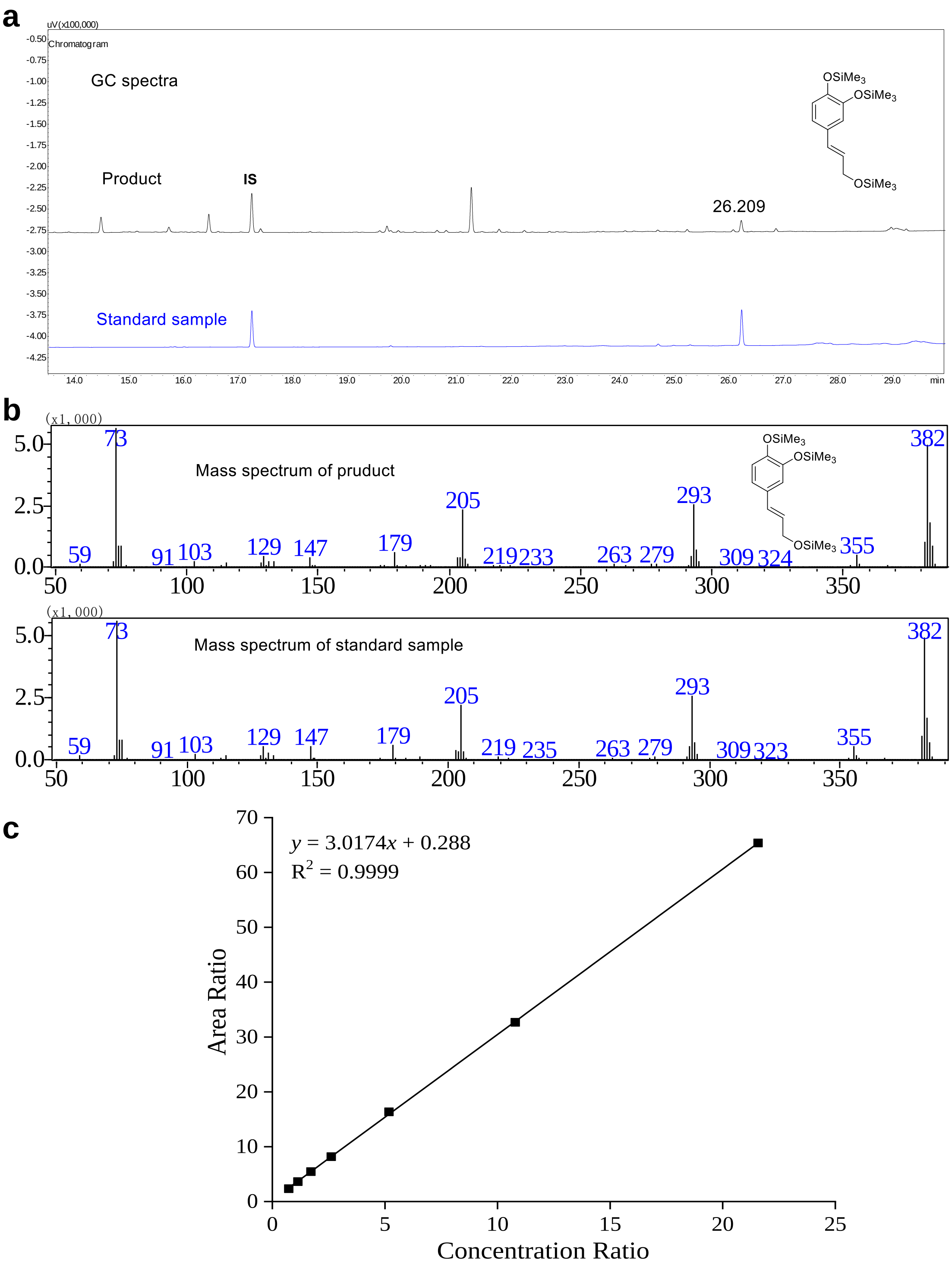


**Supplementary Figure 30.** The silylated derivative (**C1'**) of caffeyl alcohol. **a** GC spectra of product **C1'** derived from C-lignin (endocarp) depolymerization and standard sample **C1'**. **b** Mass spectra of product **C1'** derived from C-lignin (endocarp) depolymerization and standard sample **C1'**. **c** Standard curve of compound **C1'**.

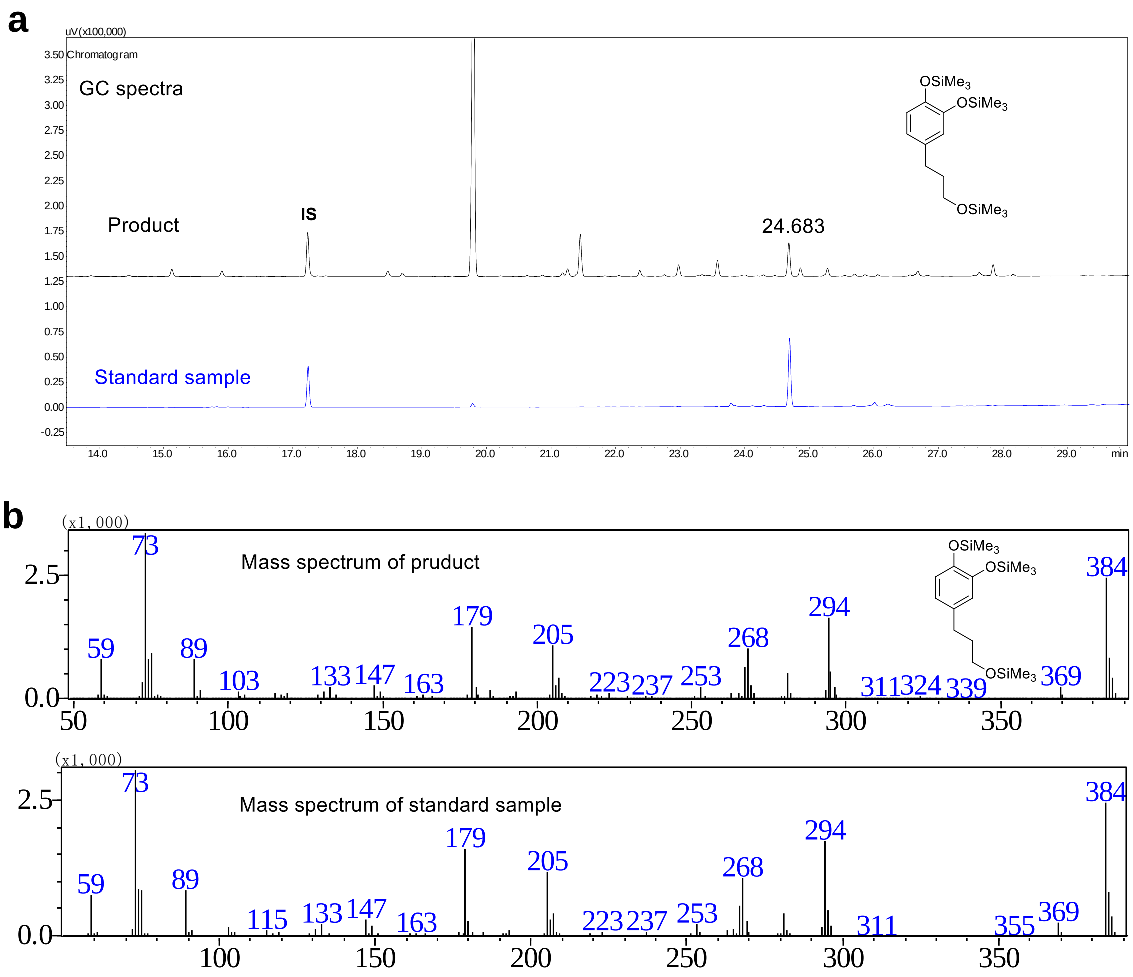


**Supplementary Figure 31.** The silylated derivative (**C2'**) of catechylpropanol. **a** GC spectra of product **C2'** derived from C-lignin (endocarp) depolymerization and standard sample **C2'**. **b** Mass spectra of product **C2'** derived from C-lignin (endocarp) depolymerization and standard sample **C2'**.

**Supplementary Note 12.** The yield of catechylpropanol **C2** compound was calculated according to our previous reported standard curve.[8]

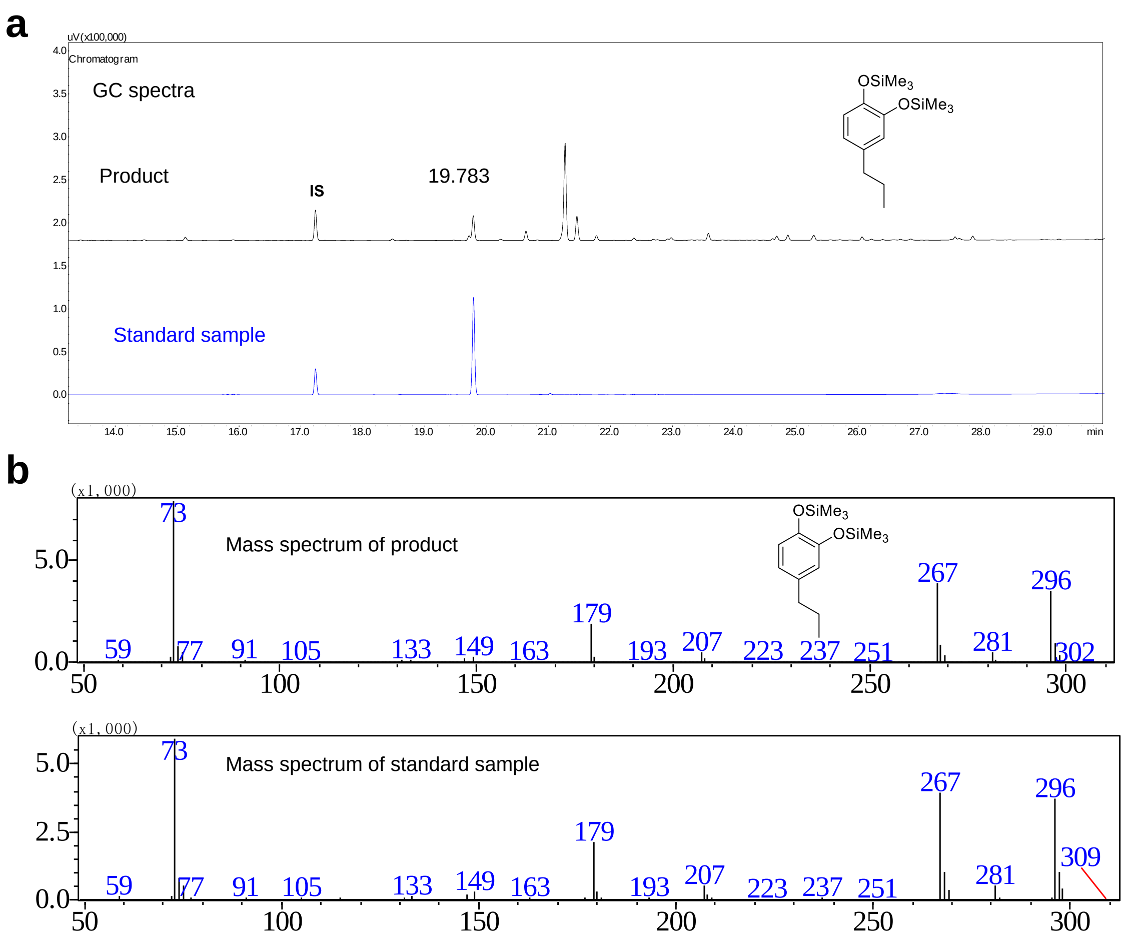


**Supplementary Figure 32.** The silylated derivative (**C3’**) of 4-propylcatechol. **a** GC spectra of product **C3’** derived from C-lignin (endocarp) depolymerization and standard sample **C3’**. **b** Mass spectra of product **C3’** derived from C-lignin (endocarp) depolymerization and standard sample **C3’**.

**Supplementary Note 13.** The yield of 4-propylcatechol **C3** compound was calculated according to our previous reported standard curve.[8]

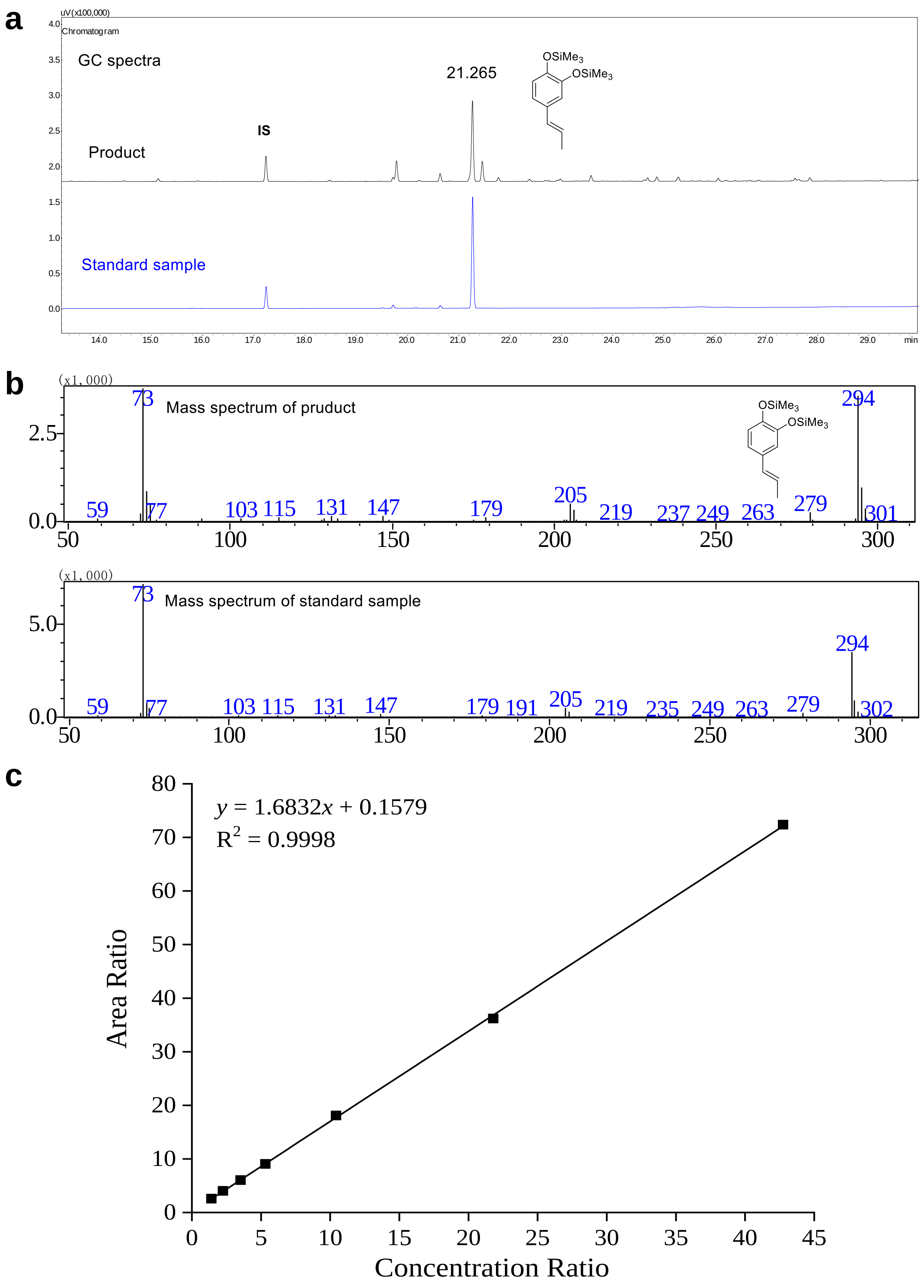


**Supplementary Figure 33.** The silylated derivative (**C4’**) of propenylcatechol. **a** GC spectra of product **C4’** derived from C-lignin (endocarp) depolymerization and standard sample **C4’**. **b** Mass spectra of product **C4’** derived from C-lignin (endocarp) depolymerization and standard sample **C4’**. **c** Standard curve of compound **C4’**.

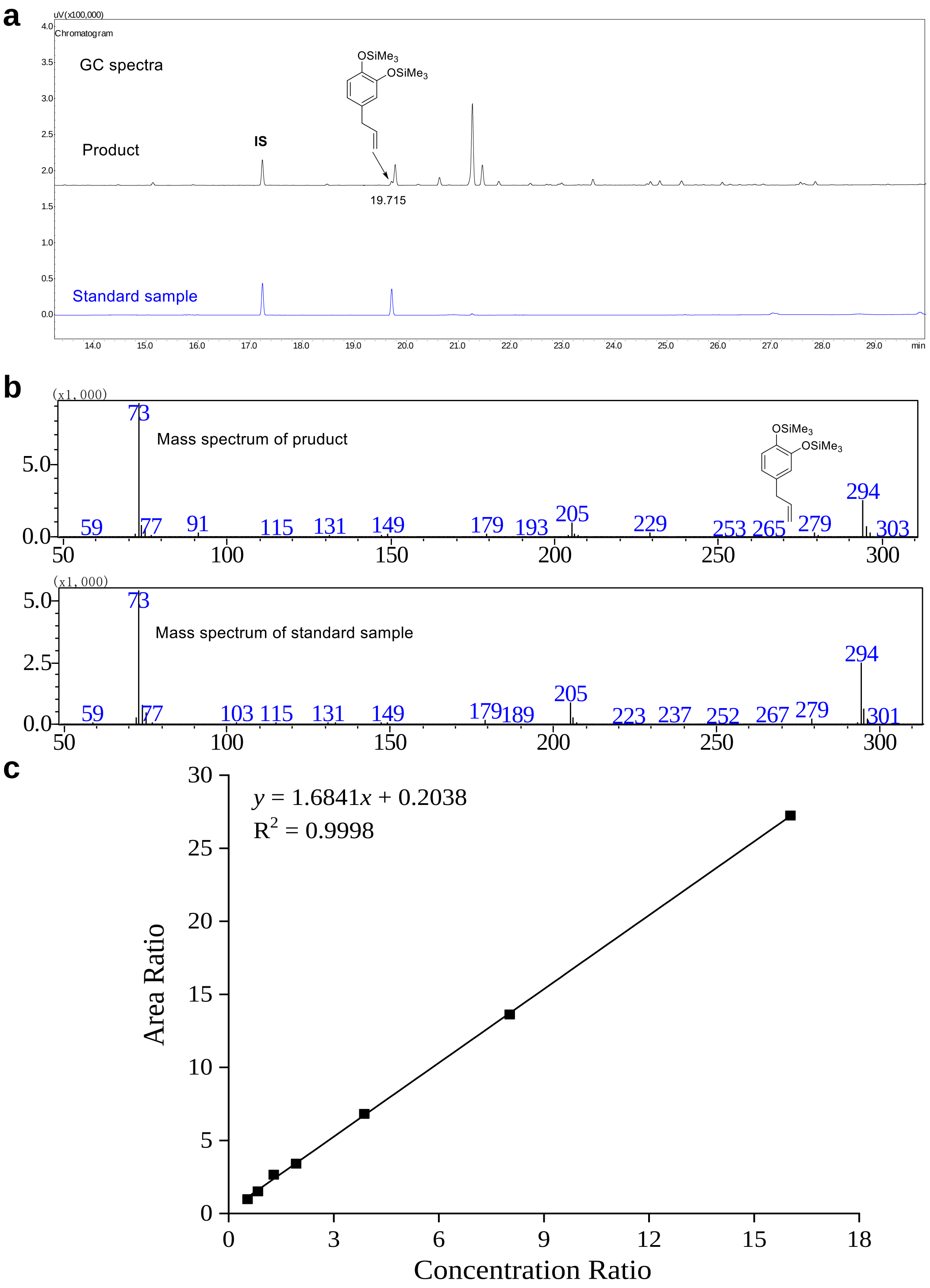


**Supplementary Figure 34.** The silylated derivative (**C5’**) of allylcatechol. **a** GC spectra of product **C5’** derived from C-lignin (endocarp) depolymerization and standard sample **C5’**. **b** Mass spectra of product **C5’** derived from C-lignin (endocarp) depolymerization and standard sample **C5’**. **c** Standard curve of compound **C5’**.

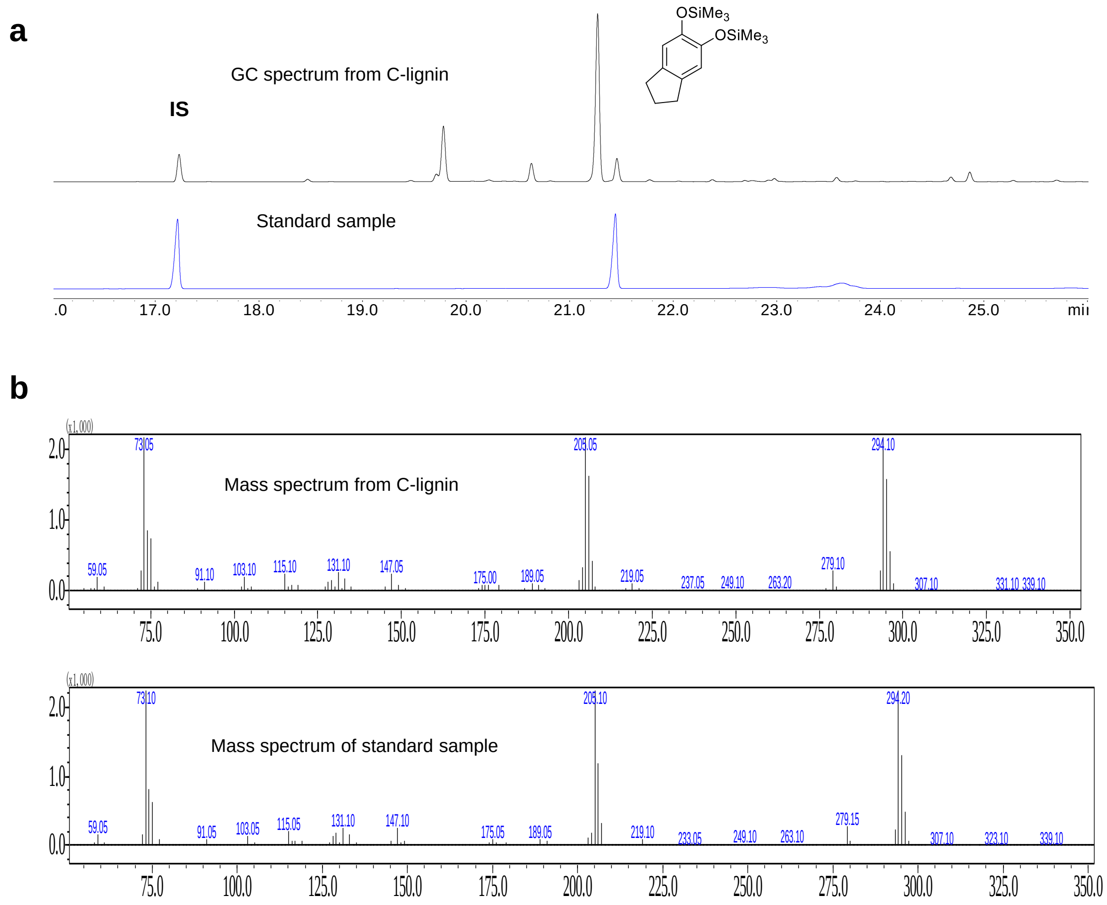


**Supplementary Figure 35.** The silylated derivative of 2,3-dihydro-1H-indene-5,6-diol. **a** GC spectra of product derived from C-lignin (endocarp) depolymerization and standard sample. **b** Mass spectra of product derived from C-lignin (endocarp) depolymerization and standard sample.

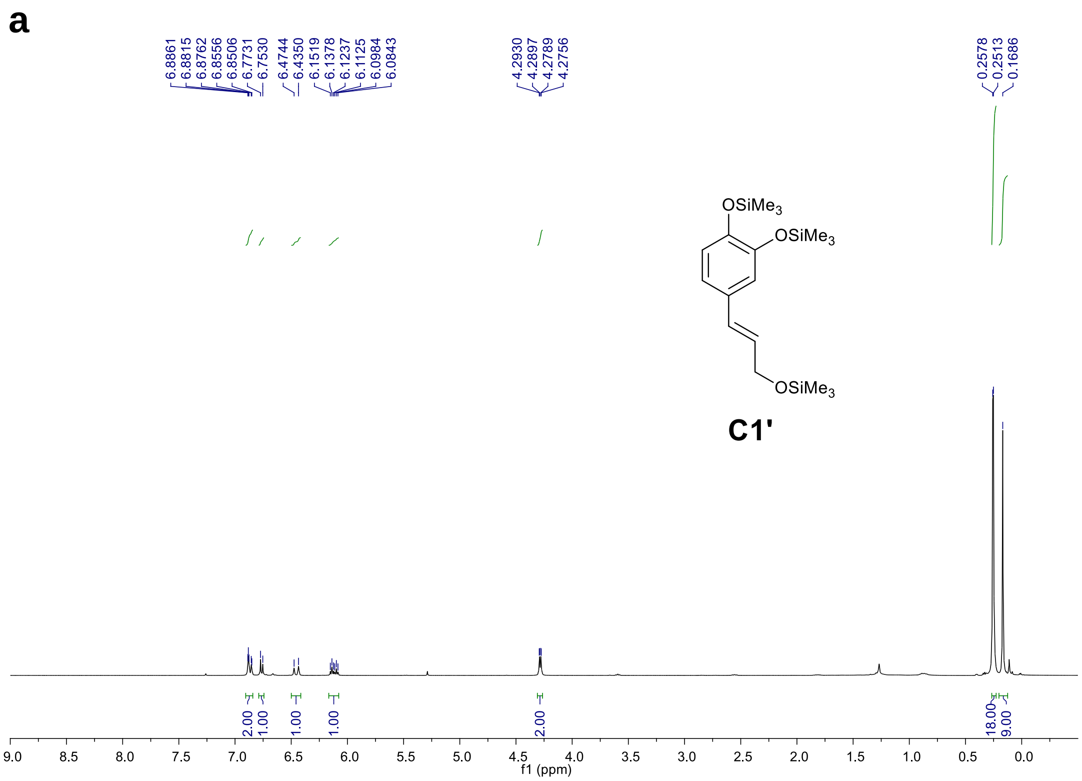


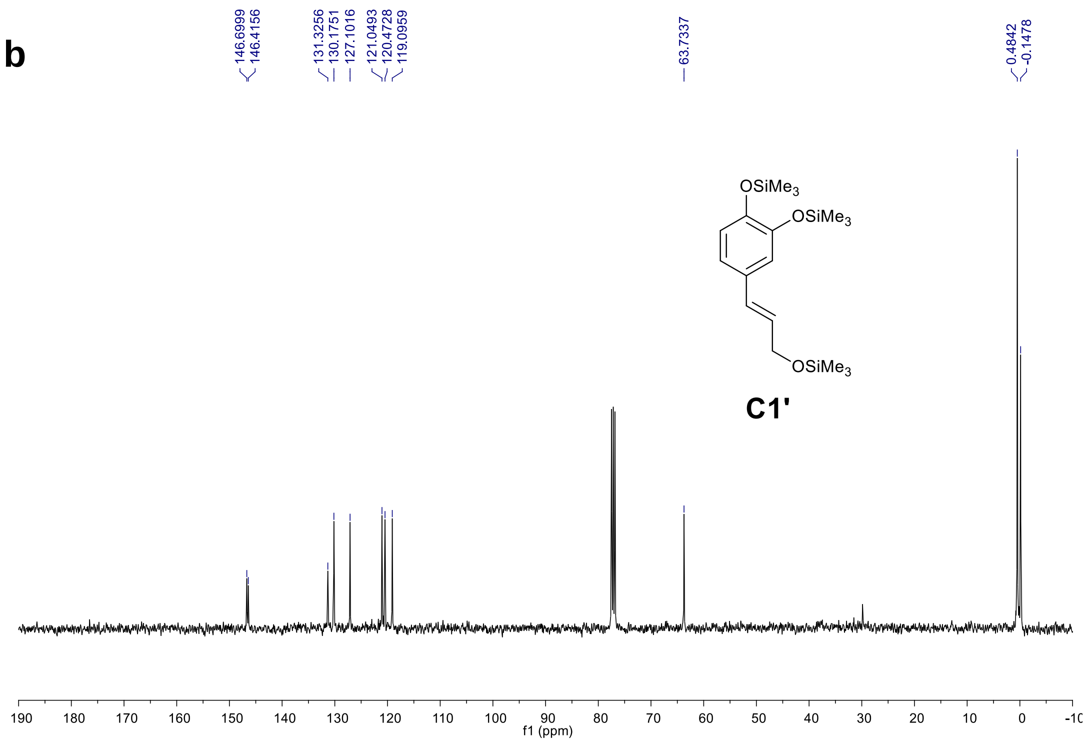


**Supplementary Figure 36.** NMR spectra of **C1’** ($CDCl_3$). **a** $^{1}H$ NMR spectrum. **b** $^{13}C$ NMR spectrum.

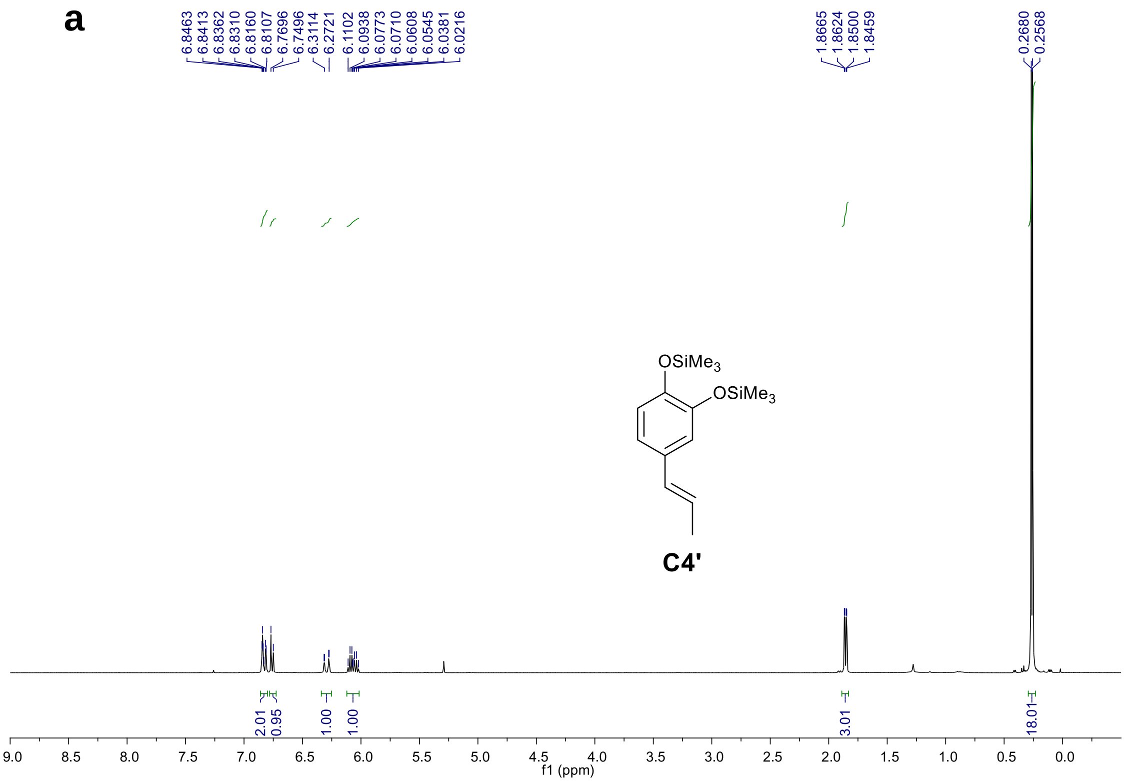


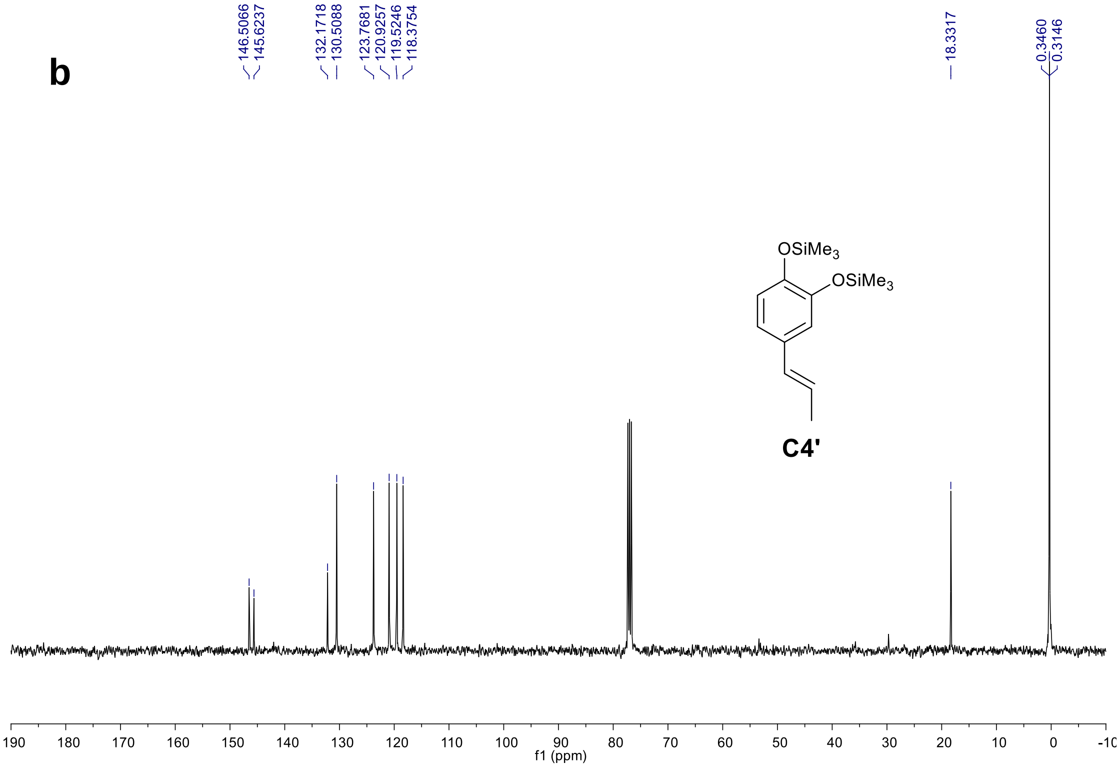


**Supplementary Figure 37.** NMR spectra of **C4’** ($CDCl_3$). **a** $^{1}H$ NMR spectrum. **b** $^{13}C$ NMR spectrum.

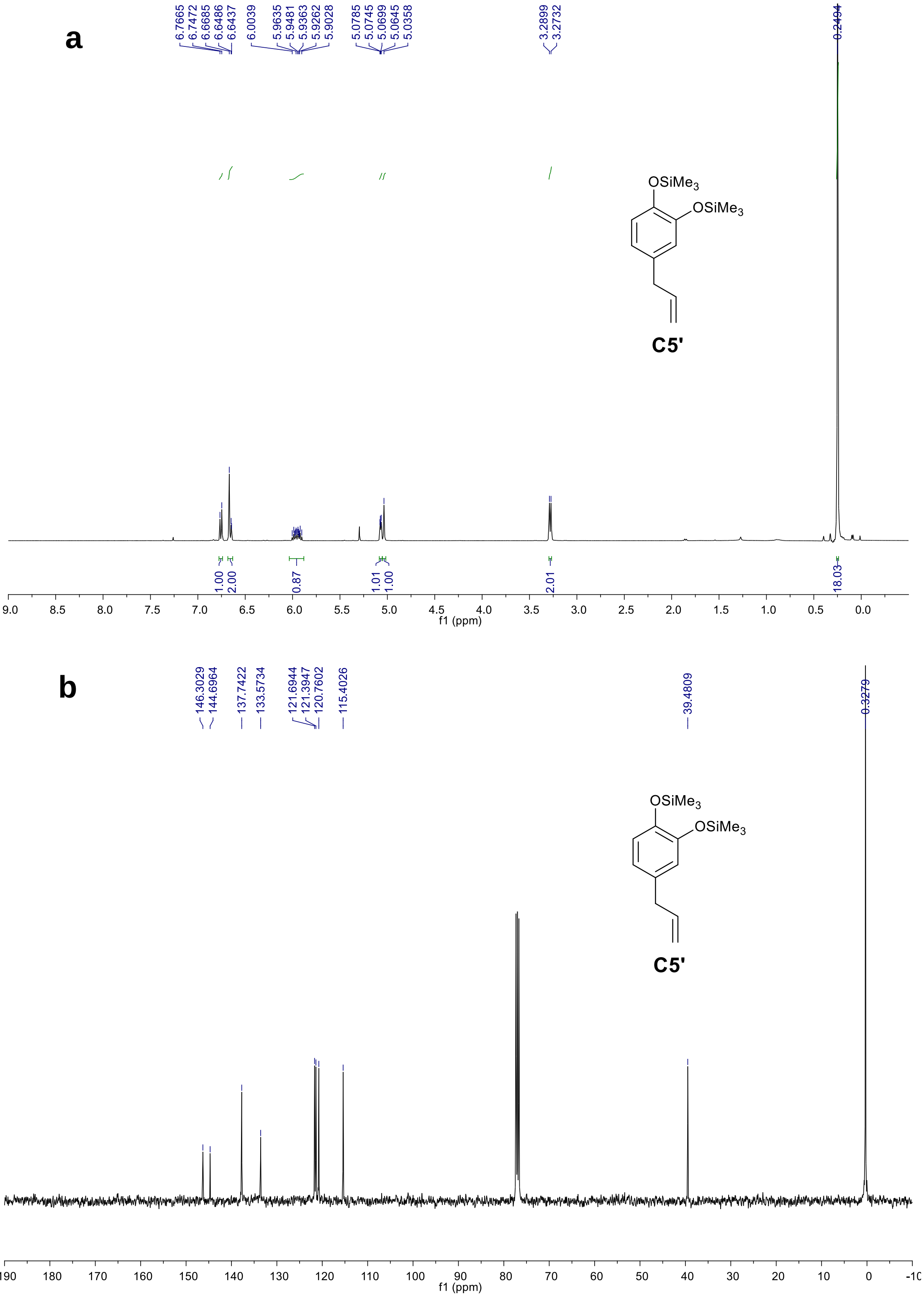


**Supplementary Figure 38.** NMR spectra of **C5’** ($CDCl_3$). **a** $^1H$ NMR spectrum. **b** $^{13}C$ NMR spectrum.

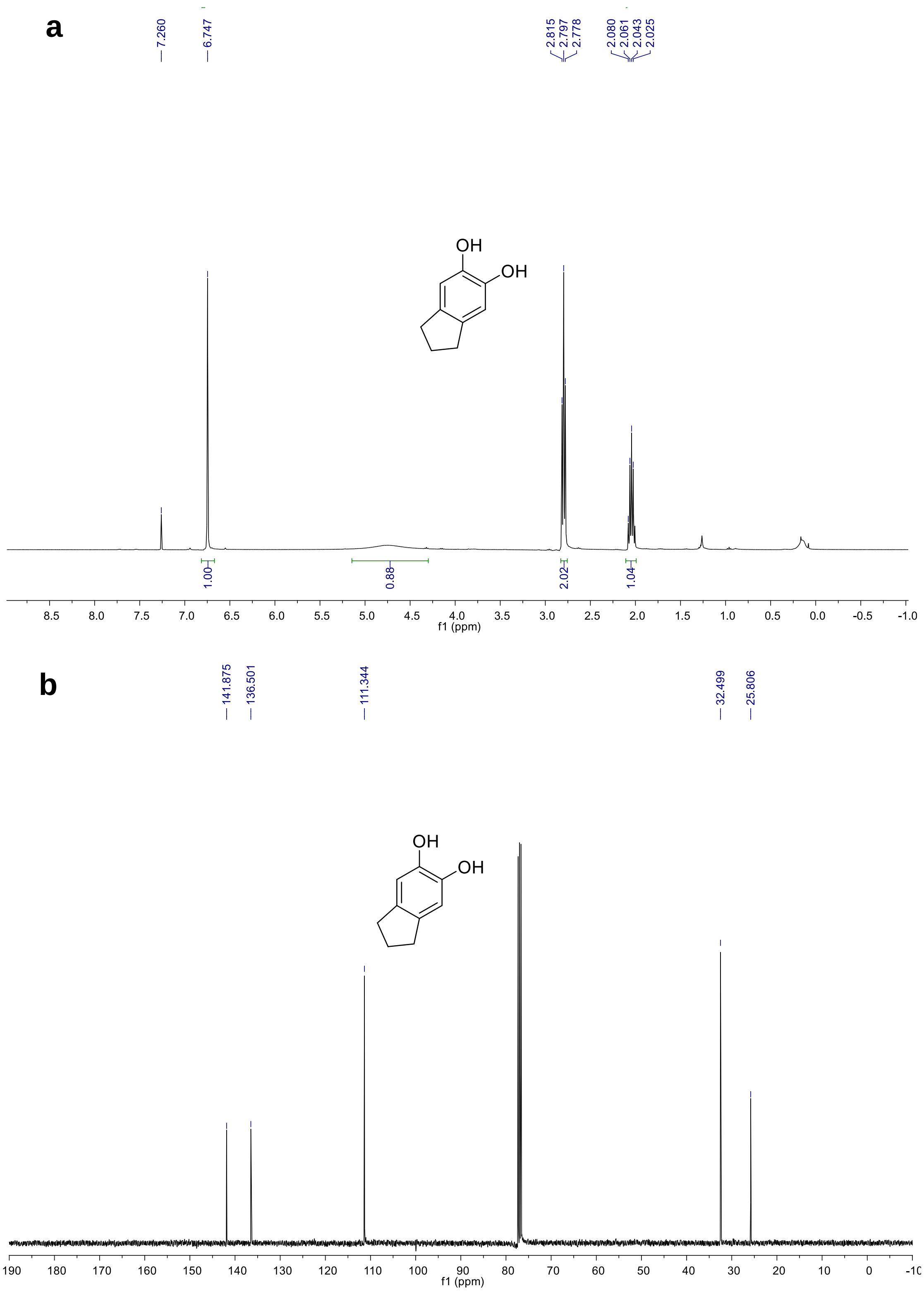


**Supplementary Figure 39.** NMR spectra of 2,3-dihydro-1H-indene-5,6-diol ($CDCl_3$). **a** $^{1}H$ NMR spectrum. **b** $^{13}C$ NMR spectrum.

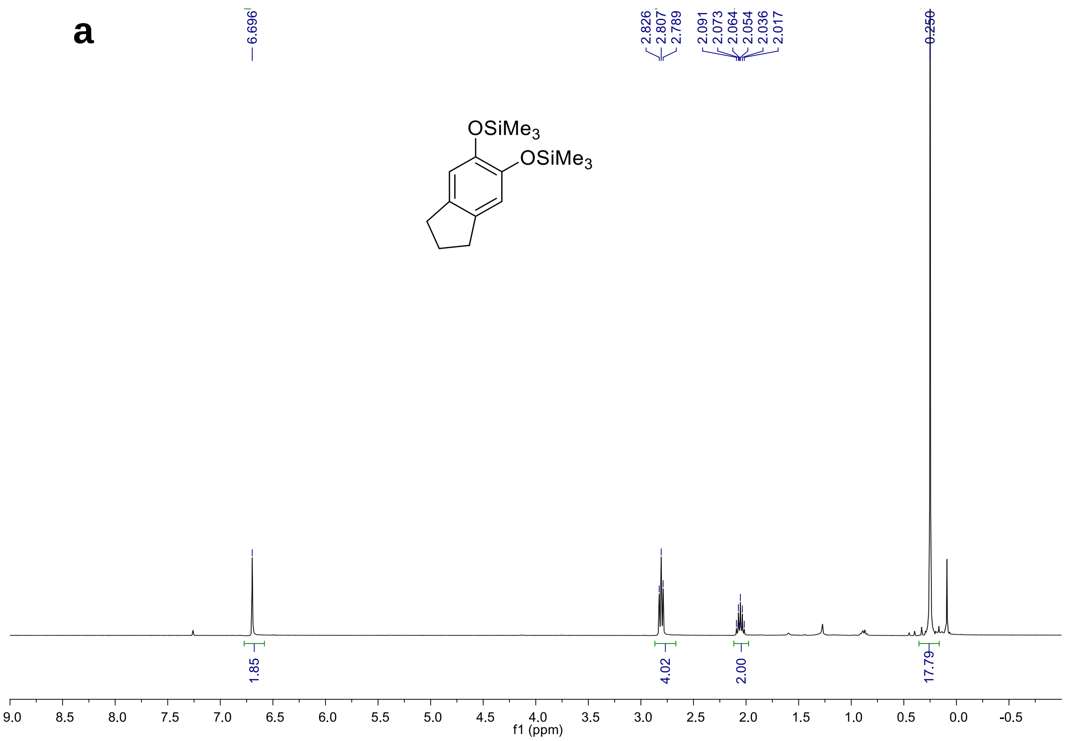


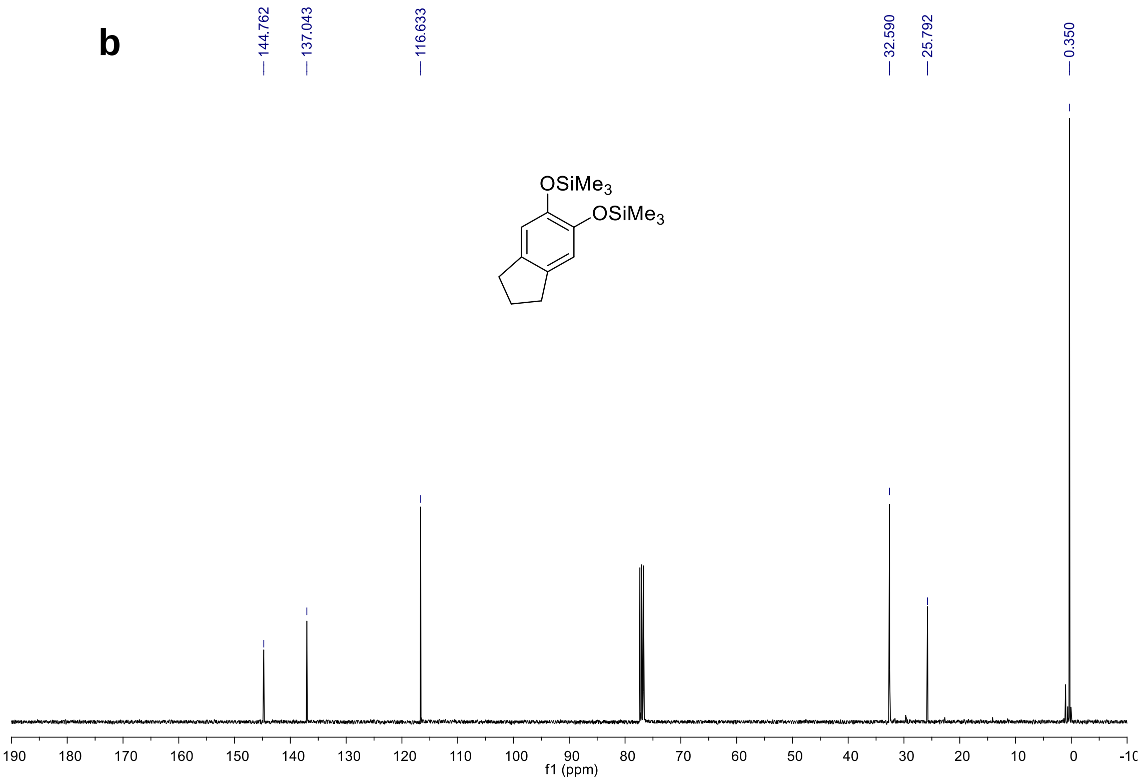


**Supplementary Figure 40.** NMR spectra of the silylated derivative of 2,3-dihydro-1H-indene-5,6-diol ($CDCl_3$). **a** $^{1}$H NMR spectrum. **b** $^{13}$C NMR spectrum.

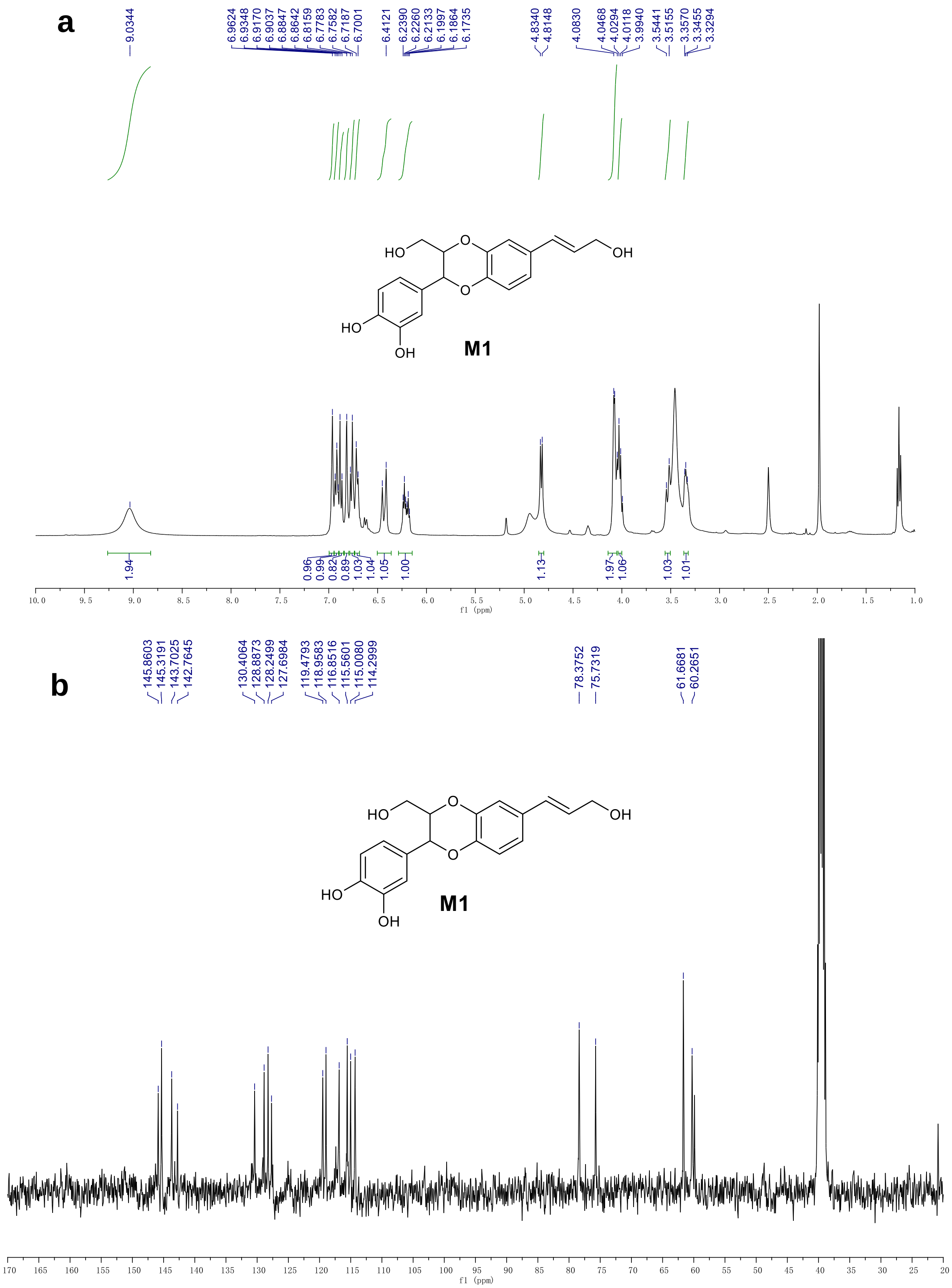


**Supplementary Figure 41.** NMR spectra of dimeric compound **M1** (DMSO-$d_6$). **a** $^1$H NMR spectrum. **b** $^{13}$C NMR spectrum.

## Supplementary References


1. Chen, W. et al, Rational Design of Single Molybdenum Atoms Anchored on N-Doped Carbon for Effective Hydrogen Evolution Reaction. *Angew. Chem. Int. Ed.* **56**, 16086-16090 (2017).
2. Ravel, B. & Newville, M. ATHENA and ARTEMIS: Interactive graphical data analysis using IFEFFIT. *Phys. Scr.* **115**, 1007-1010 (2005).
3. Sluiter, A. et al. Determination of Structural Carbohydrates and Lignin in Biomass, National Renewable Energy Laboratory (NREL), Golden, Colorado, 2008.
4. Sluiter, A. et al. Determination of ash in biomass, National Renewable Energy Laboratory (NREL), Golden, Colorado, 2008.
5. Tobimatsu, Y. et al. Coexistence but independent biosynthesis of catechyl and guaiacyl/syringyl lignin polymers in seed coats. *Plant Cell* **25**, 2587-2600 (2013).
6. Marita, J. M. et al. Structural and compositional modifications in lignin of transgenic alfalfa down-regulated in caffeic acid 3-O-methyltransferase and caffeoyl coenzyme A 3-O-methyltransferase. *Phytochemistry* **62**, 53-65 (2003).
7. Li, Y. et al. An “ideal lignin” facilitates full biomass utilization. *Sci. Adv.* **4**, eaau2968 (2018).
8. Wang, S. et al. Catechyl Lignin Extracted from Castor Seed Coats Using Deep Eutectic Solvents: Characterization and Depolymerization. *ACS Sustainable Chem. Eng.* **8**, 7031-7038 (2020).
9. Sang, D. et al. Carbodiimides as acid scavengers in aluminum triiodide induced cleavage of alkyl aryl ethers. *synthesis* **49**, 2721-2726 (2017).
